# Epigenetics underpins phenotypic plasticity of protandrous sex change in fish

**DOI:** 10.1002/ece3.8730

**Published:** 2022-03-18

**Authors:** Alyssa M. Budd, Julie B. Robins, Olivia Whybird, Dean R. Jerry

**Affiliations:** ^1^ 8001 Centre for Sustainable Tropical Fisheries and Aquaculture James Cook University Townsville Qld Australia; ^2^ 8001 Centre for Tropical Bioinformatics and Molecular Biology James Cook University Townsville Qld Australia; ^3^ Ecosciences Precinct Department of Agriculture and Fisheries Brisbane Qld Australia; ^4^ Northern Fisheries Centre Department of Agriculture and Fisheries Cairns Qld Australia; ^5^ 8001 Tropical Futures Institute James Cook University Singapore City Singapore

**Keywords:** DNA methylation, ecological adaptation, phenotypic plasticity, sex change, teleost, temperature

## Abstract

Phenotypic plasticity is an important driver of species resilience. Often mediated by epigenetic changes, phenotypic plasticity enables individual genotypes to express variable phenotypes in response to environmental change. Barramundi (*Lates calcarifer*) are a protandrous (male‐first) sequential hermaphrodite that exhibits plasticity in length‐at‐sex change between geographic regions. This plasticity is likely to be mediated by changes in DNA methylation (DNAm), a well‐studied epigenetic modification. To investigate the relationships between length, sex, and DNAm in a sequential hermaphrodite, here, we compare DNAm in four conserved vertebrate sex‐determining genes in male and female barramundi of differing lengths from three geographic regions of northern Australia. Barramundi first mature as male and later sex change to female upon the attainment of a larger body size; however, a general pattern of increasing female‐specific DNAm markers with increasing length was not observed. Significant differences in DNAm between males and females of similar lengths suggest that female‐specific DNAm arises rapidly during sex change, rather than gradually with fish growth. The findings also reveal that region‐specific differences in length‐at‐sex change are accompanied by differences in DNAm and are consistent with variability in remotely sensed sea temperature and salinity. Together, these findings provide the first *in situ* evidence for epigenetically and environmentally mediated sex change in a protandrous hermaphrodite and offer significant insight into the molecular and ecological processes governing the marked and unique plasticity of sex in fish.

## Graphical abstract

Here, we compare DNAm in four conserved vertebrate sex‐determining genes in male and female barramundi of differing lengths from three geographic regions of northern Australia. The findings reveal that region‐specific differences in length‐at‐sex change are accompanied by differences in DNAm and are consistent with variability in remotely sensed sea temperature and salinity. Together, these findings provide the first in situ evidence for epigenetically and environmentally mediated sex change in a protandrous hermaphrodite.

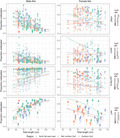

## 1 INTRODUCTION

The ability of individual genotypes to produce different phenotypes in response to environmental change, known as phenotypic plasticity, is an important driver of species resilience (Hu & Barrett, 2017; Pigliucci, 2005). In contrast to adaptive evolution, which is underpinned by random genetic mutation alone, phenotypic plasticity is often mediated by epigenetic changes (Duncan et al., 2014; Richards et al., 2010). Epigenetic changes can be defined as mitotically and/or meiotically heritable modifications that occur to the structure of DNA, rather than the nucleotide sequence, resulting in changes in phenotype in the absence of changes in genotype. Epigenetically mediated changes in phenotype exhibit a number of key attributes that differentiate them from those that are genetically underpinned: (1) they can accumulate within the lifetime of an individual, as well as intergenerationally; (2) they often arise in response to environmental stimuli (internal or external), rather than via random mutation; and (3) they can offer higher and more dynamic rates of change than genetic mutation alone (Zhang et al., 2013). In eukaryotes, the best characterized epigenetic modification is DNA methylation (DNAm), which typically involves the replacement of the 5th carbon of cytosine with a methyl group at CpG sites (i.e., where the DNA sequence is ‘CG’) but can also occur at other cytosine sites, and in other nucleotides (Jones, 2012). While it is likely that DNAm works in combination with other epigenetic mechanisms (histone modification and micro RNAs) to regulate gene expression, methods of analysis for DNAm are the most well‐established and widely used. Thus, DNAm provides an accessible and comparable measure to investigate how epigenetics underpins phenotypic plasticity in different species (e.g., Duncan et al., 2022).

Epigenetic variation can provide explanation for cases in which classical quantitative genetics, based on sequence variation alone, has been unable to explain rapid phenotypic responses to environmental change (Bossdorf et al., 2008). In fish, recent evidence suggests that population‐level epigenetic variation plays a role in generating phenotypic plasticity in wild populations. For example, in *Chrosomus eosneogaeus*, a clonal diploid fish, epigenetic variation is consistent with differences in environmental pH and occurs in the absence of genetic variation (Massicotte & Angers, 2012). Furthermore, in Atlantic salmon (*Salmo salar*), sexual maturation is mediated by DNAm (Mohamed et al., 2020), and in wild populations, high levels of epigenetic variation are thought to enable the precocious (early) maturation of small ‘sneaker’ males (Morán & Pérez‐Figueroa, 2011). Most recently, it has been shown that adaptation of shortfin mollies (*Poecilia mexicana*) to sulfidic environments is promoted through DNAm, and that these epigenetic changes are heritable (Kelley et al., 2021). These examples demonstrate how habitat‐specific DNAm can enable environmental adaptation in fish. While there are several studies that suggest epigenetic mechanisms underlie phenotypic plasticity in plants, similar studies in vertebrates are scarce (Kilvitis et al., 2017 and references therein).

**FIGURE 1:**
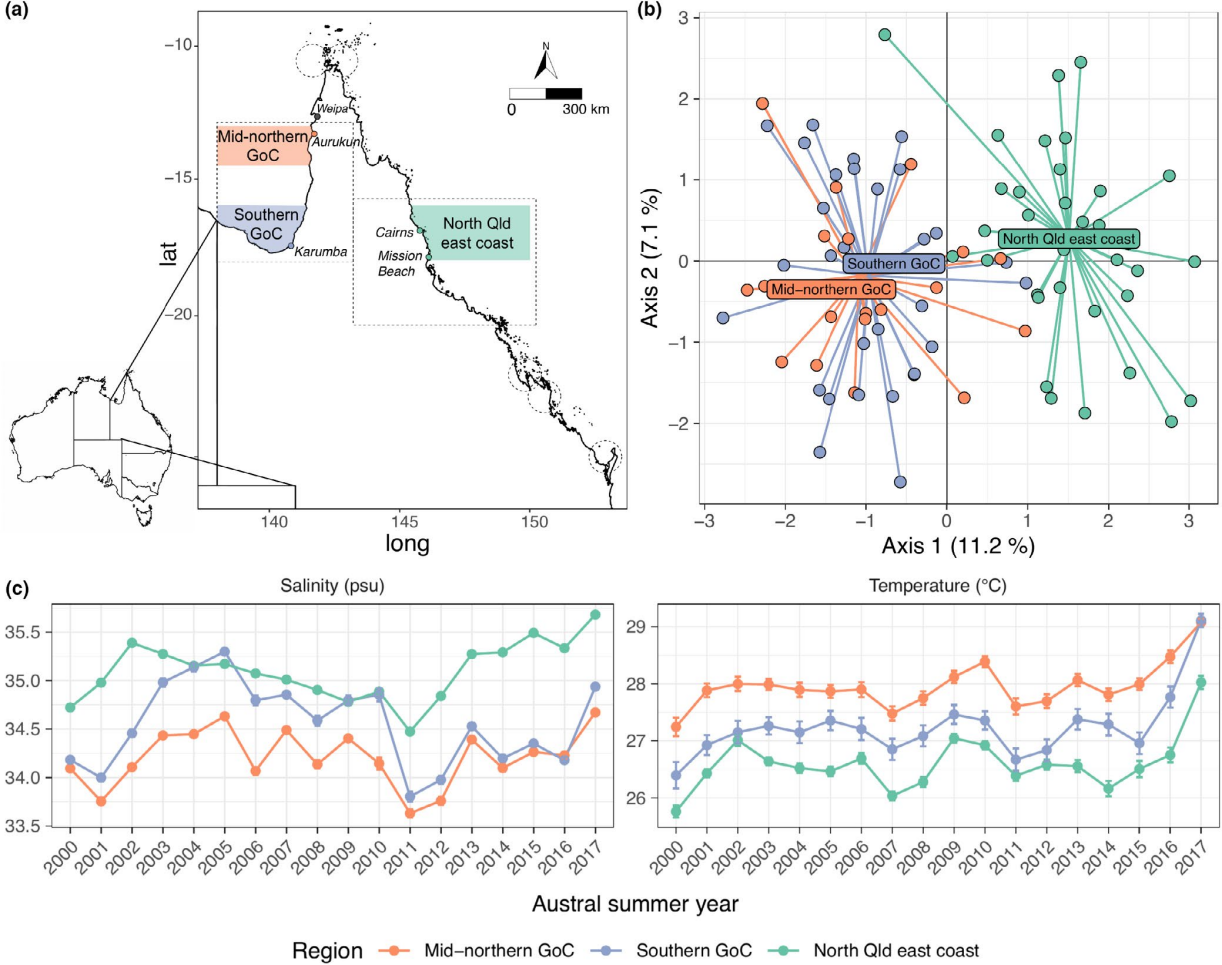
(a) Map of Queensland, Australia, showing sampling locations (colored markers) as well as distribution of the genetically distinguishable subpopulations of barramundi (*Lates calcarifer*) identified by Jerry et al. (2013; dashed circles). (b) Principal component analysis summarizing genetic diversity among the barramundi sampled and analyzed in the present study, using the same genetic markers as in the aforementioned study. (c) Average austral summer yearly (October through September) salinity and temperature estimated by the Hybrid Coordinate Ocean Model (HYCOM) for positions 8.88 km offshore of the north Queensland east coast (green), mid‐northern GoC (orange) and southern GoC (blue) barramundi catch locations. Data are represented as mean point with standard error bars

Sequential hermaphroditism presents an extreme case of epigenetically mediated, phenotypic plasticity, whereby an individual undergoes complete transition from one sex to another (Todd et al., 2016 and references therein). For example, barramundi (*Lates calcarifer*) are a protandrous (male‐first) sequential hermaphrodite fish in which sexual maturation as male and subsequent sex change to female is associated with increases in length (as anterior to posterior; Davis, 1982; Guiguen et al., 1994; Moore, 1979; Roberts et al., 2021). Not only do barramundi exhibit responsiveness to internal cues to initiate male‐to‐female sex change, but also the length at which sex change occurs differs between geographic regions, suggesting that sex is additionally responsive to external environmental cues (Davis, 1984; Loughnan et al., 2019). For example, in Australia, barramundi from the Gulf of Carpentaria (GoC) sex change at approximately 70–90 cm, compared with 85 – 100 cm in the Northern Territory (NT) and east coast of Queensland (Qld; Davis, 1982; QFMA, 1991). Furthermore, within the GoC, barramundi north of approximately 13°S (from Weipa to the northern tip of Qld; Figure 1) reach sexual maturity earlier and sex change at shorter lengths compared with barramundi from the southern GoC (i.e., Aurukun to Karumba; Figure 1), which sex change at approximately 80 cm (Davis, 1984). The underlying drivers of the differences in length‐at‐sex change in barramundi between regions are unknown, but the phenomenon has been associated with differences in genetic origin and growth rate (Davis, 1984; Roberts et al., 2021), as well as environmental factors, such as temperature and salinity (Athauda & Anderson, 2014; Budd, 2020).

The observed difference in length‐at‐sexual maturation and sex change in barramundi from different regions was previously proposed to be underpinned by genetic factors (Davis, 1984). Barramundi in Australia are considered to be composed of two genetic populations; western and eastern with a central region of admixture (Loughnan et al., 2019). Additionally, 21 genetic subpopulations have been identified, including two within the Qld region of the GoC (Figure 1; Jerry et al., 2013; Keenan, 1994). However, genetic differences are greater between the Qld east coast and GoC, rather than within the GoC where more marked differences in sexual precociousness have been observed (Davis, 1984). This suggests that differences in length and age at sexual maturation and sex change between barramundi from different geographic regions are unlikely to be explained by genetic factors alone and may be underpinned by epigenetic changes.

Sex change in many fish species has been attributed to differential DNAm of several conserved vertebrate sex‐determining genes. In barramundi, significantly higher DNAm in *dmrt1* [doublesex and mab‐3 (DM)‐related transcription factor 1] and *nr5a2* (Nuclear Receptor Subfamily 5 Group A Member 2), as well as lower DNAm in *cyp19a1a* (cytochrome P450, family 19, subfamily A, polypeptide 1a) occurs in females than that in males (Domingos et al., 2018). Similar sex‐related differences in DNAm of *dmrt1*, *cyp19a1a*, *nr5a2*, and/or *esr1* (estrogen receptor alpha) have been observed in other fish species, such as the protogynous bluehead wrasse (*Thalassoma bifasciatum*; *cyp19a1a* and *dmrt1*) (Todd et al., 2019), protogynous ricefield eel (*Monopterus albus*; *cyp19a1a*) (Zhang et al., 2013), digonic black porgy (*Acanthopagrus schlegelii*; *cyp19a1a* and *dmrt1*) (Wu et al., 2016), and gonochoristic zebrafish (*Danio rerio*; *nr5a2* and *esr1*) (Han et al., 2021; Yuan et al., 2020). Specifically, gonadal DNAm in *M*. *albus* gradually increases in *cyp19a1a* during female‐to‐male sex change, and prior to sex change, eels exhibit approximately 50% DNAm in this gene (Zhang, Zhang, et al., 2013). This suggests there is an accumulation of DNAm before the onset of sex change in *M*. *albus*. In barramundi, it is unknown whether sex‐specific differences in DNAm accumulate gradually with fish growth, or if DNAm changes occur more rapidly upon transition from male to female.

Epigenetic changes are strongly influenced by internal and external environmental cues, integrating genetic and environmental factors to produce differences in phenotype. For example, both temperature and salinity are known to affect sex in fish via changes in DNAm (see Li et al., 2017; Metzger & Schulte, 2018; Navarro‐Martín et al., 2011; Shao et al., 2014; Wang et al., 2019; Wen et al., 2014 for examples). In barramundi, maturation of the gonads and subsequent spawning is synchronized with the southwest monsoon and coincides with seasonal changes in temperature and salinity that occur just prior to its arrival (Grey, 1987; Pusey et al., 2004). Sex change is thought to commence just prior to spawning, as the testes ripen for a final time, and finish shortly after when the gonads are contracted and germ cells are multiplying (Davis, 1982). Given the intimate association between gonadal maturation and spawning, it is thought that decreases in salinity and increases in temperature concurrent with the arrival of the monsoon trigger sex change (Athauda et al., 2012; Davis, 1985). Furthermore, low salinities and high temperatures can lead to increased size and overall body condition in this species, offering greater reproductive capacity and likely leading to male‐to‐female sex change (Budd, 2020; Katersky & Carter, 2005; Partridge & Lymbery, 2008; Warner, 1988; Woo & Chiu, 1995). Thus, here, it was hypothesized that low salinities and high temperatures may be consistent with the observed geographic differences in length‐at‐sex change in barramundi, and that these phenotypic differences may be accompanied by differences in DNAm.

In this research, DNAm within amplicons covering the beginning of the first exon (start codon) and neighboring regions of four conserved vertebrate sex‐determining genes *dmrt1*, *nr5a2*, *cyp19a1a*, and *esr1* were quantified to examine the epigenetic and ecological variables governing sex change in a protandrous hermaphrodite. These sex‐determining genes are referred to throughout the manuscript as either male‐ or female‐associated, where male‐associated genes typically show higher expression and lower DNAm in male vertebrates and female‐associated genes typically show the same pattern, but in female vertebrates (Piferrer et al., 2019). Specifically, here, we (1) investigate whether changes in DNAm gradually accumulate with increasing length to become more female‐specific in barramundi; (2) compare DNAm in males and females from distinct geographic regions to identify whether differences in length‐at‐sex change are associated with differences in DNAm; and (3) use remotely sensed sea temperature and salinity estimates to explore the role of temperature and salinity as potential ecological contributors to differences in length‐at‐sex change in wild individuals.

## 2 METHODS

### 2.1 Animal collection and sampling design

Length, sex, and age data were collected as part of the Fisheries Queensland, Department of Agriculture and Fisheries (DAF) barramundi biological monitoring program from the year 2000 to 2017. Gonadal tissue samples were collected throughout the 2015 and 2016 barramundi wild harvest fishery open season (February through October) and stored in RNAlater (Thermo Fisher Scientific). Sex was determined macroscopically, whereby male and female gonads were identified by their distinct color and texture (Fisheries Queensland, 2020a). No fish undergoing testes‐to‐ovary transition were recognized in this sample set, as their identification requires histological examination (Davis, 1982). Total length (to the nearest 10 mm) was measured from the anterior‐most part of the snout to the posterior tip of the caudal fin (Fisheries Queensland, 2020a). Fish age was determined by sectioned otolith examination including increment counts, edge classification, and periodicity, as well as timing of opaque zone transformation (Fisheries Queensland, 2020b; Stuart & McKillup, 2002). Preliminary analysis revealed differences in the length at which sex change occurs (length‐at‐sex change, herein) within GoC samples; thus, the southern GoC subpopulation was split into ‘southern GoC’ (at ~16°S to land, near Karumba) and ‘mid‐northern GoC’ (at ~13°S to 14°30″S near Aurukun; Table S1; Figure 1). Only the northern Qld east coast region (at ~16°S to 18°S, near Cairns and Mission Beach; Figure 1), referred to as ‘NQ east coast’ in text, was included due to insufficient sample size south of this latitude. Stratified random sampling was used to select gonad samples for downstream DNAm analysis. For each of the three geographic regions, 1–10 gonad samples (based on sample frequency and therefore availability) from fish from each 10‐cm length category were analyzed (Figure S1). Samples did not require collection permits or animal ethics approval, as the fish were taken by recreational, charter, and commercial operators as part of usual fishing practices. Samples collected were fishery regulation capture‐dependent and restricted by minimum and maximum length limits of 58 and 120 cm, respectively.

### 2.2 Temperature and salinity data acquisition

Sea surface temperature and salinity data were obtained from the HYbrid Coordinate Ocean Model (HYCOM) and accessed via Google Earth Engine (Chassignet et al., 2009; Gorelick et al., 2017). Temperature values were validated using data from Australian Institute of Marine Science temperature loggers at available intersecting points, to achieve correlation coefficients of 0.99 where adequate coverage was available (Figure S6; AIMS, 2017). As HYCOM data points are interpolated to a standard 40 z‐levels and uniform 0.08 degrees, data were obtained for coastal regions 0.08 degrees (~8.88 km) from the specific inlets associated with reported catch locations (Fisheries Queensland, 2014). Specifically, NQ east coast temperatures are derived from coastal locations east of Snapper, Gladys, and Trinity Inlets at 16.31°S, 145.53°E; 17.50°S, 146.16°E; 16.85°S, 145.85°E, respectively. Mid‐northern GoC temperatures are derived from west of Aurukun Inlet at 13.33°S, 141.55°E, and southern GoC temperatures are derived from west of Gilbert Pocket at 16.54°S, 141.19°E. From these data, austral summer year (October through September) means were calculated for each region for use in data visualization and statistical analyses.

### 2.3 Genomic DNA extraction and microsatellite genotyping

Gonadal tissue was removed from storage in RNAlater (Thermo Fisher Scientific), washed once in PBS and dried with a KimWipe (Kimtech Science) before immediate immersion into DNA extraction buffer [100‐nM Tris‐HCl, 1.4‐M NaCl, 20‐nM EDTA, 2% cetyl trimethylammonium bromide (CTAB), and 2% polyvinylpyrrolidone (PVP)]. Genomic DNA (gDNA) was extracted following the CTAB protocol (Doyle & Doyle, 1987), including overnight digestion with Proteinase‐K followed by the addition of a phenol:chloroform:isoamyl alcohol (25:24:1) step to assist with the removal of proteins. Quantity and purity of gDNA was assessed using an ND‐1000 spectrophotometer (Nanodrop technology) based on absorbance at 260‐nm and 260/280‐nm ratio, and integrity was assessed by visualization on a 0.8% agarose gel with lambda DNA standards at 50, 20, 10, and 5 ng/µl and a 1 kb Plus DNA ladder (Thermo Fisher Scientific). Based on the findings of previous studies, it was assumed that individuals collected within the GoC were genetically similar to each other, but distinct from those originating from the NQ east coast. To validate this assumption, microsatellite genotyping was performed according to Loughnan et al. (2019), using 16 microsatellite DNA markers (loci). Genotyping was performed on the same gDNA used for BSAS, where *n* = 36 for the NQ east coast region, *n* = 32 for the southern GoC region, and *n* = 25 for the mid‐northern GoC region.

### 2.4 Bisulphite conversion of gDNA and amplico‐specific PCR

To analyze DNAm levels of the region surrounding the start codon of *cyp19a1a*, *esr1*, *dmrt1*, and *nr5a2*, a targeted bisulphite amplicon sequencing (BSAS) approach, adapted from Masser et al. (2013), was applied. These regions were selected based on our previous work, both published (*cyp19a1a* and *nr5a2*) and unpublished (*esr1* and *dmrt1*), to identify those showing significant correlations with phenotypic sex (Banh et al., 2021; Budd, 2020; Domingos et al., 2018). While the region surrounding the transcription start site is typically the most informative (Piferrer et al., 2019), not all sex‐related genes show sex‐specific DNAm patterns in amplicons near this region (e.g., *sox9*; Figure S7). Following the manufacturer's instructions, 500 ng of extracted gDNA was subject to bisulphite treatment using EZ DNA Methylation‐Gold™ (Zymo Research). Gene‐specific primers spanning the start codon of each gene were designed using MethPrimer (Li & Dahiya, 2002) with the Illumina adapter overhang nucleotide sequences added (Table S2). Gene structure diagrams indicating the positioning of the primers, CpG sites, introns, and exons of each gene were generated using the *genoPlotR* package and can be found in Figures S2 and S3. PCR amplification of these regions was achieved using Platinum^®^ Taq DNA Polymerase (Thermo Fisher Scientific) following the manufacturer's instructions. Reaction conditions were as follows: 95°C for 2 min followed by 40 rounds of 95°C for 30 s, 57.5°C for 35 s, 72°C for 40 s, with a final extension of 72°C for 10 min. PCR products were purified using Sera‐Mag SpeedBeads (GE Healthcare) prepared following Faircloth and Glenn (2014) and quantified using QuantiFluor (Promega) fluorometric nucleic acid quantitation and measured on an EnSpire Multimode plate reader (PerkinElmer).

### 2.5 NGS library preparation and DNA methylation quantification

Dual indexed libraries were generated using a Nextera XT Index Kit following the manufacturer's protocol (Illumina). Purified PCR products were indexed using limited cycle number PCR under the following reaction conditions: 95°C for 5 min followed by 12 rounds of 95°C for 30 s, 58°C for 35 s, 72°C for 40 s, with a final extension of 72°C for 10 min. Indexed amplicons were purified and quantified as described above, normalized by molarity to 4 nM and pooled into a final library. The final library was and quality checked on an Aligent TapeStation (Aligent Technologies) and quantified on a Qubit 3.0 Fluorometer (Invitrogen) for subsequent molarity determination. Libraries were then diluted to 8 pM, spiked with 20% PhiX to compensate for low base diversity in the amplicon sequencing library and loaded onto a 600 cycle V3 reagent cartridge for sequencing on an Illumina MiSeq (Illumina). FASTQ files were imported into Geneious Version 10.2.6 (Kearse et al., 2012). Paired reads were merged using BB merge paired Read Merger Version 37.25 (Bushnell et al., 2017), sequences were then trimmed based on an error probability limit of 0.05 with a maximum of one ambiguity, and resultant reads were aligned to *in silico* bisulphite converted reference sequences using the Geneious in‐built read mapper end‐to‐end read mapping and a minimum mapping quality of 30. Variants were detected using a minimum coverage of 500 reads to identify C‐T conversions in CpG positions. Because only unmethylated cytosines are converted to thymines through bisulphite treatment, at a given CpG site, the proportion of reads with C‐T variants indicates the proportion of unmethylated gene copies in the original gDNA sample. The inverse of this value gives the proportion of cytosine methylation (DNAm) for each CpG site for each gene amplicon, which was used in subsequent statistical analysis.

### 2.6 Statistical analyses

**FIGURE 2:**
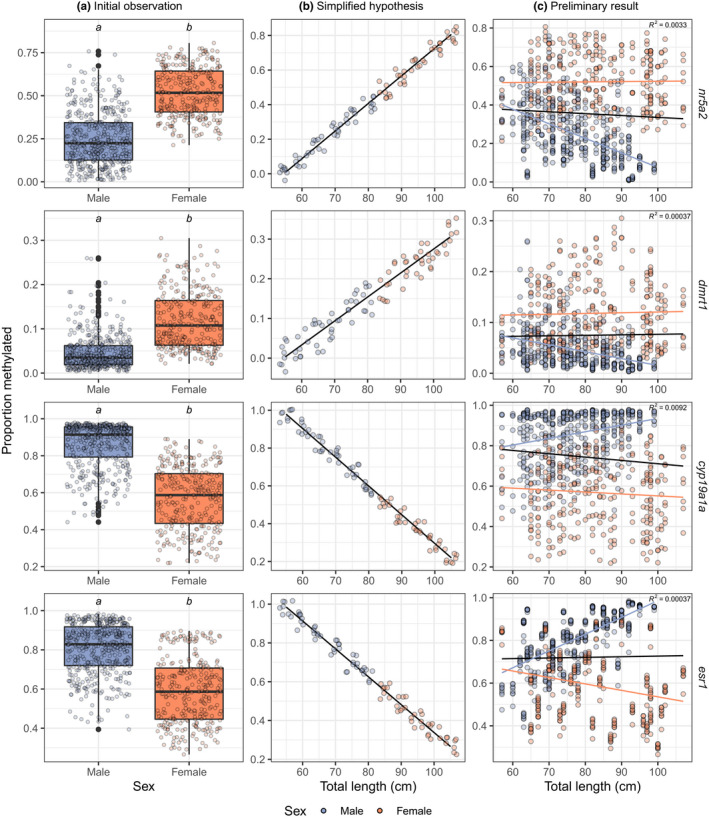
Comparison of DNA methylation levels between male and female barramundi (*Lates calcarifer*) from Queensland, Australia (all regions pooled), by amplicon. (a) Box plots demonstrating low methylation of male‐associated amplicons *dmrt1* and *nr5a2* and high methylation of female‐associated amplicons *cyp19a1a* and *esr1* and in males (blue), and the reciprocal relationship in females (orange). Letters denote significant differences between males and females resulting in the Mann–Whitney tests (*p* < .001, Table S8). (b) Hypothesized relationship between DNA methylation, length, and sex based on the results in a. (c) Preliminary generalized linear model of the relationship between DNA methylation and length (as total length, anterior to posterior), showing the overall relationship (black line), as well as the relationship for males (blue dashed line) and females (orange dashed line) separately

Statistical analyses were performed using R (version 2.15.2). Microsatellite data were analyzed using the *adegenet* package, by first converting the data using the *df2genind* function, then performing PCA analysis using the *dudi*.*pca* function (Jombart, 2008). Beta regression analyses were performed using algorithms implemented in the R package *betareg* (Cribari‐Neto & Zeileis, 2009; R Core Team, 2013). The values for the hypothesized relationship between DNAm, length, and sex (‘simplified hypothesis’; Figure 2) were calculated using the *seq* function in R‐*base* package to generate a sequence starting from the minimum to maximum proportion methylated value for any CpG within a given gene, and increments were determined by calculating largest difference between any proportion methylated value for a CpG and the minimum value for a given gene, and dividing it by 10. Length data were generated by creating a sequence between 55 and 105 cm, with increments of 5 cm, and sex data were generated using a sequence of 6 males and 5 females, respectively. Regression lines for the hypothesized relationship were generated using the *geom_line* function from *ggplot2*. Regression lines for the ‘preliminary result’ panel in Figure 2 were generated using the *geom_smooth* function, specifying the *glm* method, and R^2^ values were generated using the *stat_regline_equation* function, also from *ggplot2*. For subsequent analysis, since DNAm data generated by BSAS are proportional (bound between 0 and 1), beta regression was used to fit the data on the logit scale (Ferrari & Cribari‐Neto, 2004; Seow et al., 2012). For each gene, DNAm levels at individual CpG sites were initially modeled adjusting for sex, region, CpG site, length, and/or age with the inclusion of all possible three‐way interaction terms. To develop an appropriate model and avoid multicollinearity between fish age and length, generalized variance inflation factor (GVIF), Pearson's correlation coefficients, Bayesian information criterion (BIC), and pseudo‐adjusted coefficients of determination (pseudo *R* ^2^) values were calculated. While GVIF analysis did not indicate a problem with multicollinearity (Table S3), regression analysis revealed a significant relationship between age and length (*p* < .001; Figure S5). With the exception of *dmrt1*, all BIC values for models including length (but not age) were more preferable to those including age (but not length; Table S4). Therefore, based on the regression results and BIC values calculated, length was retained in the subsequent analyses, and age was excluded (Table S5, Table S6). The inclusion of length, but not age, in the model is supported by a recent analysis of barramundi from Western Australia, which identified that sex change is more closely related to length than age (Roberts et al., 2021).

A chi‐square test of goodness of fit based on deviance was performed to determine the effect of each factor in the model and identify interaction terms that were nonsignificant for all amplicons, which were subsequently dropped from the model. The resulting model used for all amplicons included six interaction terms, five of which included sex. Given that sex had significant interactions with all other factors (Table S5), the data were subset by sex (male and female) to enable greater resolution in the resulting beta regression models (Table S6). To account for multiple testing, a Benjamini‐Hochberg FDR correction was applied (Benjamini & Hochberg, 1995). The final model for each sex within each gene predicted DNAm based on the three remaining factors (CpG, region, and length) and included one significant interaction term (region: length). The final models explained 20%–80% of the total variance in DNAm, depending on the gene and sex (Table S6). Post hoc evaluation of the differences in the mean response between geographic regions (holding other predictors at their means) was performed through linear hypothesis testing (*linearHypothesis* function, *car* package; Fox et al., 2013). Binomial GLMs for fitting length frequency distributions were performed using general linear models (*glm* function, R‐base *stats* package) using Fisheries Queensland data years 2000 to 2017. Temperature and salinity data were analyzed using one‐way ANOVA (*aov* function in the R‐base *stats* package) followed by pairwise comparisons with Bonferroni corrections (*glht* function, *multcomp* package).

## 3 RESULTS

### 3.1 Confirmation of genetic structure

PCA of microsatellite genotyping provided a summary of the genetic diversity among the sampled individuals in this study (Figure 1). The results confirmed the assumption that individuals from the mid‐northern and southern GoC (as defined here) are of a single genetic origin and are genetically differentiated from the NQ east coast (Figure 1b). This assumption was based on previous research defining the population genetic structure of barramundi in Australia (Jerry et al., 2013; Loughnan et al., 2019).

### 3.2 Significant differences in modeled sea temperature and salinity between regions

Analysis of variance for HYCOM salinity and temperature data revealed significant associations between region and yearly mean salinity [*F* (2, 51) = 29.69, *p* < .001] and temperature [*F* (2, 51) = 34.84, *p* < .001]. Pairwise comparisons between the three regions more specifically revealed significant differences in both salinity and temperature between all three regions *(p* < .05), where the mid‐northern GoC exhibits the lowest salinity and highest temperature values, the NQ east coast exhibits the opposite (highest salinity and lowest temperature values), and the southern GoC exhibits intermediate salinity and temperature (Figure 1c).

### 3.3 DNA methylation is sex‐specific, but the relationship between methylation, length, and sex is not as predicted

Analysis of the gonads of wild barramundi revealed that males and females exhibit highly divergent DNAm in all amplicons of the four sex‐associated genes examined (Table S8, Figure 2). Specifically, higher DNAm levels were observed in the female‐associated amplicons *cyp19a1a* and *esr1* in males, and higher DNAm levels in male‐associated amplicons *dmrt1* and *nr5a2* in females (Table S8 and Figure 2a). On average, female‐associated amplicons *cyp19a1a* and *esr1* were 33% and 24% more methylated in males, and male‐associated amplicons *dmrt1* and *nr5a2* were 7% and 29% less methylated in male compared to that in females (Table S8 and Figure 2a). Thus, male‐specific DNAm was characterized by high DNAm in *cyp19a1a* and *esr1* and low DNAm in *dmrt1* and *nr5a2*. Reciprocally, female‐specific DNAm was characterized by comparatively low DNAm in *cyp19a1a* and *esr1* and comparatively high DNAm in *dmrt1* and *nr5a2* (Table S8 and Figure 2a).

Preliminary regression analysis revealed substantial variation in DNAm with fish length in all four amplicons of the sex‐associated genes examined, indicating a more complex relationship between DNAm, length, and sex than was initially hypothesized (Figure 2b,c). This was due to substantial overlap in size classes between males and females, where DNAm levels are more strongly predicted by sex than length (Figure 2c, Table S5). This led to high variability in DNAm levels at a given length, with obvious differences between males and females (Figure 2c). Where DNAm and length were modeled with both sexes together, the direction of this relationship (i.e., positive or negative) was consistent with our hypothesis of increasing female‐specific DNA methylation levels with length for two of the four sex‐associated gene amplicons (*dmrt1* and *cyp19a1a*). However, in all cases, the association was very weak (as indicated by low *R* ^2^ values, Figure 2b,c). Where the sexes are modeled separately, females largely follow the hypothesized trend (positive for male‐associated amplicons, negative for female‐associated amplicons, with large variance) and males do not. Thus, the relationship between DNAm and sex was, for all four sex‐associated amplicons, directionally divergent (Figure 2c). Analysis using beta regression including all covariates (length, sex, region, and CpG site) revealed significant interactions between sex and all other factors (Table S5). This result confirmed that the relationship between DNAm and length, sex, region, and CpG site is different between the sexes, and as such, the data were subset by sex in the subsequent analyses (Table S6).

### 3.4 DNA methylation in males becomes more male‐specific with increasing length

**FIGURE 3:**
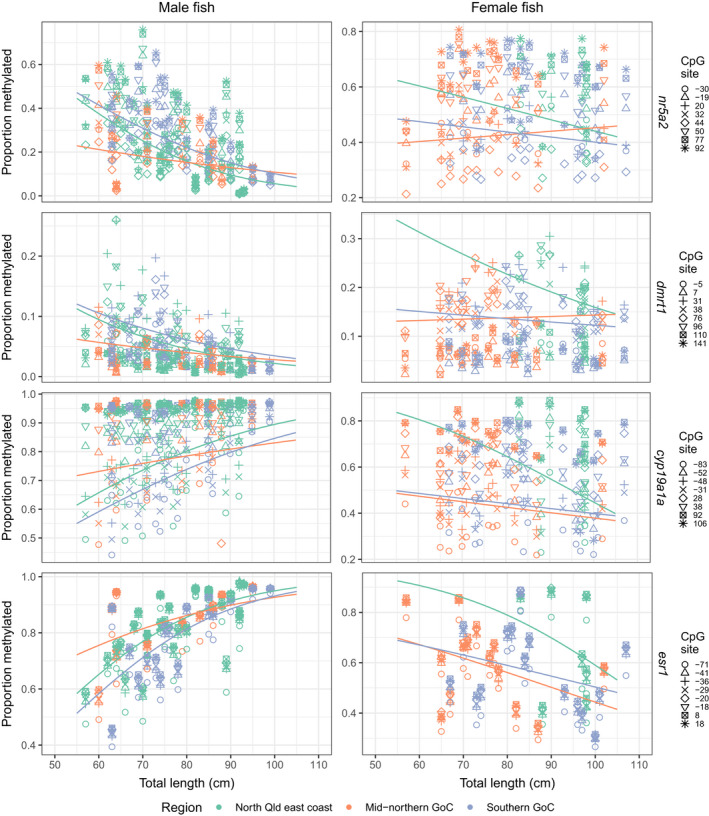
Proportion of DNA methylation in male (left column) and female (right column) barramundi (*Lates calcarifer*) explained by length (as total length in centimeters) and CpG site (as base pair position) for amplicons of male‐associated amplicons *nr5a2* and *dmrt1* and female‐associated amplicons *cyp19a1a* and *esr1*. Fitted curves correspond to beta regression with logit link for three regions in Queensland, Australia (indicated by color). Curves were evaluated at varying lengths, and the CpG with intercept value closest to the average value is shown. Model: Proportion methylated ~CpG site + region + total length + region: total length with logit link

Beta regression modeling confirmed that barramundi exhibit a significant relationship between DNAm levels and length in all four amplicons of key sex‐related genes examined. However, as length increases, males typically show an increase in male‐specific DNAm, rather than female‐specific. For example, DNAm of female‐associated amplicons *cyp19a1a* and *esr1* increased with length in males (Figure 3). Similarly, DNAm of male‐associated amplicons *dmrt1* and *nr5a2* decreased with length in males (Figure 3). This was in contradiction to the expected increase in DNAm of male‐associated amplicons with increasing length initially hypothesized to lead to size‐related male‐to‐female sex change (Figure 2b).

### 3.5 DNA methylation in females generally becomes more female‐specific with increasing length

In most cases, DNAm in females became more female‐specific with increasing length. However, it is unlikely that any DNAm changes in females contribute to sex change as these individuals have already changed sex. Specifically, for females from all regions, beta regression modeling revealed that DNAm of female‐associated amplicons became more female‐specific as length increased (Figure 3). In contrast, for male‐associated amplicons, only females from the mid‐northern GoC exhibited increases in female‐specific DNAm as length increased (Figure 3, Figure 2b). These observations are consistent with the predicted increase in female‐specific DNAm with growth. However, these cumulative changes in DNAm occurred following sex change (i.e., in females), rather than prior to sex change (i.e., in males). Therefore, the present results do not provide support for gradual, cumulative changes in DNAm leading to male‐female sex change in this species.

### 3.6 Mid‐northern GoC barramundi demonstrate differential DNA methylation

Mid‐northern GoC males demonstrated differential DNAm compared to males from both other regions sampled. More specifically, individual hypothesis testing revealed that mid‐northern GoC males had significantly different DNAm levels compared to males from the NQ east coast and southern GoC, which were not significantly different from each other (Table S7). Mid‐northern GoC males demonstrated more male‐specific DNAm in smaller length classes, becoming more similar to males from the southern GoC and NQ east coast in larger length classes (Figure 3, Table S6). The trend was similar for the relationship between DNAm and increasing age (Figure S6). In females, DNAm was more variable between both amplicon and geographic region comparisons compared to males. Individual hypothesis testing revealed significant differences between NQ east coast region and both GoC regions for *cyp19a1a*, but only between NQ east coast and the southern GoC for *esr1* (Table S7). In NQ east coast females, DNAm in female‐associated amplicons (*cyp19a1a* and *esr1*) were higher overall, and changes with length were of greater effect size compared to females from the mid‐northern and southern GoC (Figure 3, Table S6). While DNAm levels in male‐associated amplicons (*nr5a2* and *dmrt1*) were also higher in females from the NQ east coast, the direction of the relationship between DNAm and length followed the inverse relationship for females from the mid‐northern GoC compared with the other regions (i.e., was positive rather than negative; Figure 3). In summary, NQ east coast females tend towards higher levels of DNAm than GoC females in all amplicons and mid‐northern GoC females exhibit a directionally divergent relationship between DNAm and length for male‐associated amplicons.

### 3.7 Mid‐northern GoC barramundi demonstrate differences in length‐ and age‐at‐sex change

**FIGURE 4:**
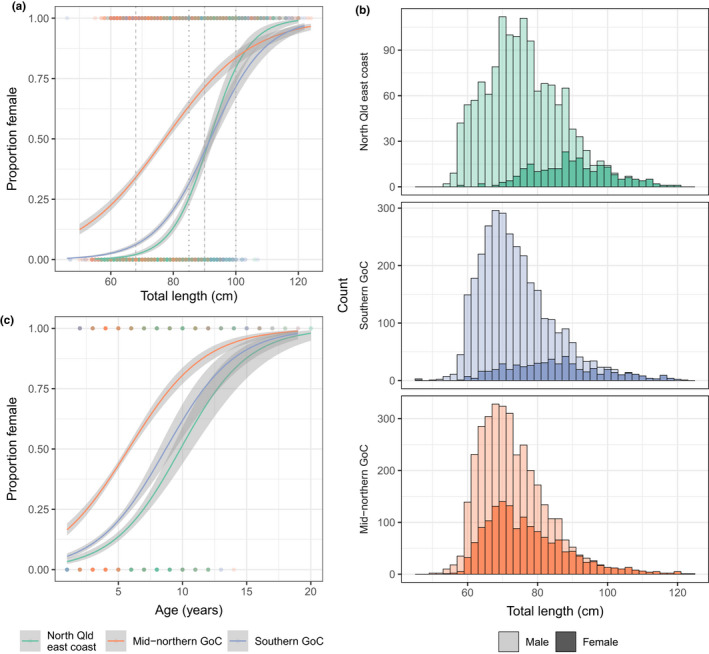
Analysis of length (as total length, anterior to posterior), sex, and age in barramundi (*Lates calcarifer*) from the north Qld east coast (*n* = 1247; green), mid‐northern Gulf of Carpentaria (GoC; *n* = 2319; orange), and southern GoC (*n* = 3169; blue) >50 cm, Australia, collected between 2000 and 2017. (a) Fitted binomial GLM (solid lines) with standard error (gray shading) showing predicted proportion of females at a given length for each region. Gray dashed and dotted vertical lines indicate previously reported size ranges for sex change in the northern GoC (68–90 cm) and north Qld east coast (85–100 cm), respectively. (b) Length frequency distribution showing the total number of male (light color shades) and female (dark color shades) barramundi of each 10‐cm size class sampled for length, age, and sex in each region. (c) Fitted binomial GLM (solid lines) with standard error (gray shading) showing predicted proportion of females at a given age for each region

Binomial GLM confirmed the relationship between length and sex, with length significantly predicting sex [*b* = 0.098, *z*(6761) = 29.333, *p* < .001; where b indicates the beta value, or coefficient, and *z* is the *z*‐statistic]. The model further demonstrated that region was a significant predictor, with the mid‐northern GoC more strongly differentiated from the NQ east coast [*b* = 2.394, *z*(6761) = 23.687, *p* < .001] than the southern GoC [*b* = 0.26, *z*(6761) = 2.649, *p* < .01]. Visualizing the model revealed a clear shift towards females of smaller length classes from the mid‐northern GoC, indicating that barramundi from this region exhibit a smaller length‐at‐sex change than those from the southern GoC and NQ east coast, in addition to exhibiting differential DNAm (Figure 4a,b). Binomial GLM of the relationship between age and sex demonstrate that age is a significant predictor of sex [*b* = 0.342, *z*(6761) = 20.81, *p* < .001], where the mid‐northern GoC is again more strongly differentiated from the NQ east coast [*b* = 1.654, *z*(6761) = 18.270, *p* < .001], compared to that of the southern GoC [*b* = 0.337, *z*(6761) = 3.564, *p* < .001]. A higher proportion of females at younger ages was observed for the mid‐northern GoC (Figure 4c).

## 4 DISCUSSION

Barramundi exhibit phenotypic plasticity in length‐at‐sex change between geographic regions and sex‐specific DNAm in key sex genes (Davis, 1984; Domingos et al., 2018). Here, the analysis of DNAm in two male‐ and two female‐associated genes revealed that: (1) A general trend of increasing female‐specific DNAm patterns with increasing length was not observed in barramundi, despite the occurrence of male‐to‐female sex change at larger body lengths, and (2) region‐specific differences in length‐at‐sex change are consistent with differences in DNAm. Notably, these differences occurred between two regions of the GoC that represent a single genetic subpopulation, indicating that epigenetics underpins phenotypic plasticity in length‐at‐sex. Additionally, differences in DNAm and length‐at‐sex change between the mid‐northern GoC, southern GoC, and NQ east coast were consistent with differences in remotely sensed sea temperature and salinity estimates, suggesting these may be ecological drivers of epigenetic and phenotypic variation in sex.

### 4.1 No general trend of increasing female‐specific DNAm patterns with length in barramundi

The occurrence of male‐to‐female sex change at larger body lengths in barramundi lead to the expectation of a general increase in female‐specific DNAm patterns with length. It was hypothesized that these increases in female‐specific DNAm (in sex‐associated genes) would eventually reach a threshold level and trigger male‐to‐female sex change. However, a trend of female‐specific DNAm with increasing length was not observed for males from any region in any of the four sex genes examined. In females, DNAm generally became more female‐specific with increasing length, most consistently for female‐associated genes. In other words, an increase in sex‐specific DNAm, rather than a general increase in female‐specific DNAm, with length was observed. Therefore, sex change cannot be attributed to an increase in female‐specific DNAm, as this trend was only observed in individuals that had already undergone the sex change process (i.e., it was only observed in females). As such, when examined as single‐point, group‐level data, it does not appear that gradual changes in DNAm over time is what leads to male‐to‐female sex change, at least in the four gene regions examined here.

From these data alone, it cannot be excluded that, at the individual level, a gradual accumulation in DNAm over time may occur. However, the disparity between DNAm levels in males and females of similar lengths and the observation that large males exhibit more male‐specific DNAm with increasing length suggest that the initially predicted gradual accumulation is unlikely. Instead, female‐specific DNAm may arise rapidly upon male‐to‐female sex change, rather than gradually over time with growth. Indeed, male‐to‐female sex change in barramundi occurs in less than 17 days (Guiguen et al., 1994). This rapid change in phenotypic sex provides further indication that sex‐specific changes in DNAm occur equally rapidly, and a possible explanation for the sizable and significant differences in DNAm between males and females are described here.

An alternate explanation for the observed increase in sex‐specific DNAm with length is that DNAm is the consequence, rather than the cause of sex change in fish. Due to the inverse relationship between DNAm and gene expression in vertebrates, including many cases of *cyp19a1a* and *dmrt1* in fish, it is generally accepted that changes in DNAm lead to changes in gene expression and phenotypic sex (Anastasiadi et al., 2018; Piferrer et al., 2019 and references therein). However, here, it was found that DNAm in both sexes became more sex‐specific with increasing length, despite the well‐established relationship between length and sex in barramundi (i.e., males are small and females are large—Davis, 1982; Guiguen et al., 1994; Moore, 1979; Roberts et al., 2021). DNAm is strongly impacted by a genes’ transcriptional state in fish, and other organisms (McGaughey et al., 2014), suggesting that changes in DNAm may arise due to sex‐specific transcriptional activity, rather than (or in addition to) DNAm being the cause of changes in transcription of sex‐related genes. As such, DNAm as a result of transcription may be involved in the maintenance of gonadal phenotype. In addition, while DNAm can cause changes in hormone production and hormone receptor expression, hormones can also cause changes in DNAm, for example, through the regulation of their associated enzymes (DNMTs; Baumbach & Zovkic, 2020). In barramundi specifically, estrogen treatment resulted in decreased DNAm and increased expression of *cyp19a1a*, as well as early male‐to‐female sex change (Banh et al., 2021). It remains unknown whether changes in DNAm occur prior to or following changes in gene expression and natural hormone production, which lead to sex change in fish.

Time‐course analysis of the gonads before, during, and after sex change in individuals would provide increased insight into how sex‐specific DNAm arises, and precisely how these relate to changes in sexual phenotype. Previous work in wild‐caught bluehead wrasse (Todd et al., 2019) has significantly increased understanding of DNAm and gene expression in transitional individuals (i.e., in fish actively undergoing female‐to‐male sex change). However, for barramundi, the identification of transitional individuals cannot be macroscopically determined and usually requires destructive sampling followed by histological examination of the gonad (Davis, 1982). Furthermore, the short timeframe in which sex change occurs, as well as a fishery closure during the spawning season, means that transitional individuals are rarely captured in the wild. For example, Moore (1979) reported a frequency of 1.4% of transitional individuals. In captive barramundi, however, nondestructive gonadal cannulation samples are routinely taken for the assessment of reproductive status of broodstock. Appropriate preservation of these samples would allow for the analysis of gonadal DNAm and gene expression in individuals during the stages of adult sexual development (i.e., male, transitional, and female), and enable better insight into the molecular events preceding and succeeding sex change in fish.

The observation that males exhibit increasingly more male‐specific DNAm with increasing length suggests that not all barramundi undergo male‐to‐female sex change (as was first suggested by Moore, 1979). Previous work in both wild and cultured barramundi has shown that while the average body size (as length and/or weight) of males is smaller than that of females of the same age, there is substantial overlap in size distributions between the sexes (Guiguen et al., 1994; Lim et al., 1986; Yue et al., 2012). This substantial overlap was also observed in the present study. Collectively, these results suggest that there are factors in addition to body size, which lead to sex‐specific DNAm and male‐to‐female sex change (or not). For example, there may be social factors involved in the maintenance of male and/or development of female barramundi [as first proposed by Guiguen et al. (1994)], as is the case in many species of protogynous grouper (e.gLiu & Sadovy de Mitcheson, 2011; Mackie, 2000, 2003). Similarly, density is known to affect DNAm and female‐to‐male sex change in protogynous rice field eels (Zhang, Zhang, et al., 2013). The effects of social factors such as density and sex ratio on DNAm and sex change in a protandrous fish, particularly in mass and group spawning species, are yet to be investigated.

### 4.2 Region‐specific differences in length‐at‐sex change are reflected in DNA methylation of males

While previous reports have shown differences in length‐at‐sex change between barramundi from the Qld east coast and GoC more broadly (QFMA, 1991), the present analysis revealed similarities in length‐at‐sex change between the north Qld east coast and southern GoC (with both regions occurring slightly south of 16°S; see Figure 1 and Figure 4a). Furthermore, previously reported differences in length‐at‐sex change within the GoC were only reported *north* of 13°S (Davis, 1984). However, the present results showed significant differences in length‐at‐sex change between two regions of the GoC that occur *south* of Davis’ 13°S (Figure 1). While abrupt variation in length‐at‐sex change was previously proposed to infer underlying genetic drivers (Davis, 1984), this new evidence (reported here) indicates that variation in length‐at‐sex is more clinal than previously thought and offers support for epigenetically mediated sex change in barramundi. Differences in length‐at‐sex change occurred within a single genetic subpopulation (i.e., within the GoC), and genetic analyses showed no evidence for substantial genetic dissimilarity between the southern and mid‐northern GoC regions as defined here (Figure 1; Jerry, 2014; Keenan, 1994; Loughnan et al., 2019; Shaklee & Salini, 1985). Thus, the present results suggest that the phenotypic differences in length‐at‐sex change observed between the southern and mid‐northern GoC are primarily underpinned by epigenetic and not genetic differences.

Male barramundi from the mid‐northern GoC demonstrated differential DNAm compared with males from both other regions sampled, as well as smaller lengths‐at‐sex change. The shorter lengths‐at‐sex change suggest a similar but less marked sexual precociousness (including early sexual maturation and sex change) for the mid‐northern GoC than what has been previously observed for the far northern GoC (Davis, 1984). Sexual precociousness has been associated with differences in DNAm of *cyp19a1a* and other genes in animals as diverse as humans and grouper (Guo et al., 2021; Stueve et al., 2014). In barramundi, sexual precociousness may be underpinned by a shift in DNAm trajectory towards shorter length classes, resulting in sex change at shorter lengths. Here, mid‐northern GoC males exhibited more marked differences in DNAm at shorter lengths compared with other regions, suggesting that this predicted shift is present but truncated at the lower end (due to legal size limits for barramundi in Queensland; i.e., individuals <55 cm and >120 are absent from the data). In addition to truncated DNAm data, these size limits meant that growth curves could not be accurately calculated. Future work measuring changes in DNAm in barramundi below the legal size limit will allow for region‐specific examination of the potential association between growth rate and early sexual maturation, as well as early sex change (Roberts et al., 2021; Warner, 1988).

### 4.3 Differences in length‐at‐sex change are consistent with variation in salinity and temperature

The effect of temperature on sex in fish has been documented in over 60 species, with many laboratory‐reared individuals exhibiting associated differences in DNAm (Budd et al., 2015, and references therein). In barramundi specifically, temperature experiments have demonstrated an association between high temperatures and male‐to‐female sex change (Athauda et al., 2012). These temperature‐induced differences have recently been shown to be accompanied by changes in DNAm, and the upregulation of signaling pathways thought to be involved in the transduction of temperature cues (Budd, 2020; Castelli et al., 2020). Here, it was observed that the mid‐northern GoC exhibits significantly higher temperatures, lower salinities, shorter lengths‐at‐sex change, and differential DNAm compared to that in the southern GoC and NQ east coast regions. These results suggest that the positive association between environmental temperature and male‐to‐female sex change occurs in natural environments, in addition to laboratory experiments. To our knowledge, the observations here present the first *in situ* investigation of ecological variables, epigenetics, and sequential sex change in wild fish.

In addition to temperature and salinity, there may be alternative, additive and/or related mechanisms leading to the differences in length‐at‐sex change and DNAm between regions observed here. For example, the stress hormone cortisol has been identified as the link between social factors and protogynous (female‐to‐male) sex change in bluehead wrasses, leading to increased DNAm and decreased expression of *cyp19a1a* (Goikoetxea et al., 2017; Todd et al., 2019). Similarly, in some gonochoristic fish, high‐temperature exposure leads to an increase in cortisol production, decrease in *cyp19a1a* expression, and subsequent testis development (e.g., Hattori et al., 2009; Hayashi et al., 2010). It is likely that exposure to cold temperatures causes thermal stress and reduced growth in barramundi (Newton et al., 2013), which may lead to differences in length‐at‐sex change. Growth and body condition are also highly likely to lead to differences in fecundity and the timing of male‐to‐female sex change in protandrous species, including barramundi (Roberts et al., 2021; Warner, 1988). In barramundi, freshwater flow (e.g., Robins et al., 2006) and food availability (e.g., Russell et al., 2015) have marked effects on growth rate, which is known to lead to differences in the timing of male‐to‐female sex change in this species (Roberts et al., 2021). In the case of temperature, the effect on male‐to‐female sex change may therefore be indirect (e.g., through changes in hormone levels and/or growth rate), direct (e.g., via cellular transduction of temperature cues), or both. Further research that aims to better understand the relationships between DNAm, hormonal shifts, sex, and environmental variables, including experimental and functional studies, will yield greater insight into environmentally mediated phenotypic plasticity in length‐at‐sex change in barramundi and other species. This information may also contribute to understanding how GoC barramundi populations will respond to fluctuations in environmental conditions under a changing climate.

### 4.4 Implications for fisheries management

Barramundi support important recreational, commercial, and indigenous fisheries across the species’ entire distribution, throughout the Indo‐West Pacific from the Arabian Gulf, across south‐east Asia to northern Australia and Papua New Guinea (Grey, 1987). Protandrous sex change presents a unique challenge for fisheries management, necessitating both upper and lower legal catch length limits to protect a proportion of male and female spawners, respectively (i.e., a gauntlet fishery). Protandrous sex change also increases the complexity of population models needed to assess stock status (Benvenuto et al., 2017; Campbell et al., 2017). Current fisheries modeling for barramundi, specifically in Queensland, Australia, relies on the assumption that sex ratios are unchanging over time and space, and that the data collected by Davis (1982, 1984) on sex (including maturity and fecundity) at length nearly 40 years ago are temporally representative (Campbell et al., 2017). Given the demonstrated plasticity of sex change in barramundi within Queensland identified here, it is likely that fisheries modeling could be improved through the use of region‐specific data (see Streipert et al., 2019). DNAm and related measures are potentially highly useful molecular markers for population demographics in fisheries modeling, for example, age and total lifespan (Mayne et al., 2019, 2021). The results presented here, among others, suggest that DNAm and other epigenetic measurements could also be used to predict sexual development (including sexual differentiation, maturity, and change) and likely fecundity (Heydenrych et al., 2021; Piferrer et al., 2019). Further work on the identification and development of epigenetic indicators of sex in commercially important fish species will enable better representation of environmentally mediated biological processes in fisheries modeling, stock assessments, and refined management strategies, particularly for sex‐changing species.

## 5 CONCLUSION

In sequentially hermaphroditic fish, sex change is often associated with the attainment of a minimum body length. More recently, sex change has also been associated with epigenetic changes. Here, we present observations of epigenetic (as DNAm) and phenotypic variation in length‐at‐sex change in a protandrous hermaphrodite. Our results suggest the rapid onset of sex‐specific DNAm upon male‐to‐female sex change, as DNAm in males and females of similar lengths is disparate. Additionally, DNAm in males becomes more male‐specific (rather than more female‐specific) with increasing length, despite the occurrence of male‐to‐female sex change at larger body lengths. Further analysis revealed region‐specific relationships between length and DNAm, which were accompanied by differences in length‐at‐sex change. The greatest epigenetic and phenotypic differences were observed between regions that were genetically indistinct, providing evidence for epigenetically underpinned phenotypic plasticity. Finally, the observed epigenetic and phenotypic differences were consistent with ecological differences in sea temperature and salinity, suggesting that these may be direct, or indirect, ecological drivers of DNAm and length‐at‐sex in barramundi. Together, these findings provide the first *in situ* investigation of epigenetics, ecology, and sequential sex change in a protandrous fish. More broadly, the research provides a unique example of how differential DNAm of trait‐of‐interest genes is associated with phenotypic plasticity in a natural environment.

## CONFLICT OF INTEREST

The authors declare that they are not aware of any competing interests.

## AUTHOR CONTRIBUTIONS

**Alyssa M. Budd:** Conceptualization (equal); Data curation (lead); Formal analysis (lead); Funding acquisition (equal); Investigation (lead); Methodology (lead); Project administration (equal); Resources (equal); Validation (lead); Visualization (lead); Writing – original draft (lead); Writing – review & editing (lead). **Julie B. Robins:** Conceptualization (supporting); Data curation (supporting); Formal analysis (supporting); Investigation (supporting); Methodology (supporting); Resources (equal); Validation (supporting); Visualization (supporting); Writing – original draft (supporting); Writing – review & editing (supporting). **Olivia Whybird:** Data curation (equal); Investigation (supporting); Methodology (supporting); Project administration (supporting); Resources (equal); Validation (supporting); Writing – original draft (supporting); Writing – review & editing (supporting). **Dean R. Jerry:** Conceptualization (equal); Formal analysis (supporting); Funding acquisition (equal); Investigation (supporting); Methodology (supporting); Project administration (equal); Resources (equal); Supervision (lead); Validation (supporting); Visualization (supporting); Writing – original draft (supporting); Writing – review & editing (supporting).

### OPEN RESEARCH BADGES

This article has earned an Open Data Badge for making publicly available the digitally‐shareable data necessary to reproduce the reported results. The data is available at https://doi.org/10.5061/dryad.dbrv15f32; https://github.com/dr‐budd/eco_epigen.

## Supporting information

Supplementary Material

## Data Availability

Data and code for the DNA methylation analyses can be found at https://github.com/dr‐budd/eco_epigen. Raw sequence data and metadata are archived in the Dryad data repository at https://doi.org/10.5061/dryad.dbrv15f32.

## References

[ece38730-bib-0001] Anastasiadi, D. , Esteve‐Codina, A. , & Piferrer, F. (2018). Consistent inverse correlation between DNA methylation of the first intron and gene expression across tissues and species. Epigenetics & Chromatin, 11(1), 37. 10.1186/s13072-018-0205-1 29958539 PMC6025724

[ece38730-bib-0002] Athauda, S. , & Anderson, T. (2014). Effect of temperature and salinity on sex inversion in Asian Seabass (*Lates calcarifer*): Relationship with plasma sex steroids concentration and aromatase activity of gonad and brain. Aquaculture Research.

[ece38730-bib-0003] Athauda, S. , Anderson, T. , & de Nys, R. (2012). Effect of rearing water temperature on protandrous sex inversion in cultured Asian Seabass (*Lates calcarifer*). General and Comparative Endocrinology, 175(3), 416–423. 10.1016/j.ygcen.2011.11.040 22155035

[ece38730-bib-0004] Australian Institute of Marine Science (AIMS) (2017). AIMS Sea Water Temperature Observing System (AIMS Temperature Logger Program). 10.25845/5b4eb0f9bb848accessed. 21‐May‐2021.

[ece38730-bib-0005] Banh, Q. Q. , Guppy, J. L. , Domingos, J. A. , Budd, A. M. , Pinto, R. C. , Marc, A. F. , & Jerry, D. R. (2021). Induction of precocious females in the protandrous barramundi (*Lates calcarifer*) through implants containing 17β‐estradiol‐effects on gonadal morphology, gene expression and DNA methylation of key sex genes. Aquaculture, 539, 736601. 10.1016/j.aquaculture.2021.736601

[ece38730-bib-0006] Baumbach, J. L. , & Zovkic, I. B. (2020). Hormone‐epigenome interactions in behavioural regulation. Hormones and Behavior, 118, 104680. 10.1016/j.yhbeh.2020.104680 31927018

[ece38730-bib-0007] Benjamini, Y. , & Hochberg, Y. (1995). Controlling the false discovery rate: A practical and powerful approach to multiple testing. Journal of the Royal Statistical Society: Series B (Methodological), 57(1), 289–300.

[ece38730-bib-0008] Benvenuto, C. , Coscia, I. , Chopelet, J. , Sala‐Bozano, M. , & Mariani, S. (2017). Ecological and evolutionary consequences of alternative sex‐change pathways in fish. Scientific Reports, 7(1), 1–12. 10.1038/s41598-017-09298-8 28831108 PMC5567342

[ece38730-bib-0009] Bossdorf, O. , Richards, C. L. , & Pigliucci, M. (2008). Epigenetics for ecologists. Ecology Letters, 11(2), 106–115. 18021243 10.1111/j.1461-0248.2007.01130.x

[ece38730-bib-0010] Budd, A. M. (2020). Epigenetic effects of temperature on sex change in barramundi, Lates calcarifer (Doctor of Philosophy (PhD)). James Cook University.

[ece38730-bib-0011] Budd, A. M. , Banh, Q. Q. , Domingos, J. A. , & Jerry, D. R. (2015). Sex control in fish: Approaches, challenges and opportunities for aquaculture. Journal of Marine Science and Engineering, 3(2), 329–355. 10.3390/jmse3020329

[ece38730-bib-0012] Bushnell, B. , Rood, J. , & Singer, E. (2017). BBMerge–accurate paired shotgun read merging via overlap. PLoS One, 12(10), e0185056. 10.1371/journal.pone.0185056 29073143 PMC5657622

[ece38730-bib-0013] Campbell, A. B. , Robins, J. , & O'Neill, M. (2017). Assessment of the barramundi (*Lates calcarifer*) fishery in the Southern Gulf of Carpentaria. Queensland.

[ece38730-bib-0014] Castelli, M. A. , Whiteley, S. L. , Georges, A. , & Holleley, C. E. (2020). Cellular calcium and redox regulation: The mediator of vertebrate environmental sex determination? Biological Reviews, 95(3), 680–695. 10.1111/brv.12582 32027076

[ece38730-bib-0015] Chassignet, E. , Hurlburt, H. , Metzger, E. J. , Smedstad, O. , Cummings, J. , Halliwell, G. , Bleck, R. , Baraille, R. , Wallcraft, A. , Lozano, C. , Tolman, H. , Srinivasan, A. , Hankin, S. , Cornillon, P. , Weisberg, R. , Barth, A. , He, R. , Werner, F. , & Wilkin, J. (2009). US GODAE: Global ocean prediction with the HYbrid Coordinate Ocean Model (HYCOM). Oceanography, 22(2), 64–75. 10.5670/oceanog.2009.39

[ece38730-bib-0016] Cribari‐Neto, F. , & Zeileis, A. (2009). Beta regression in R.

[ece38730-bib-0017] Davis, T. L. O. (1982). Maturity and sexuality in barramundi, *Lates calcarifer* (Bloch), in the Northern Territory and south‐eastern Gulf of Carpentaria. Marine and Freshwater Research, 33(3), 529–545. 10.1071/MF9820529

[ece38730-bib-0018] Davis, T. L. O. (1984). A population of sexually precocious barramundi, *Lates calcarifer*, in the Gulf of Carpentaria, Australia. Copeia, 144–149. 10.2307/1445045

[ece38730-bib-0019] Davis, T. L. O. (1985). Seasonal changes in gonad maturity, and abundance of larvae and early juveniles of barramundi, *Lates calcarifer* (Bloch), in Van Diemen Gulf and the Gulf of Carpentaria. Marine and Freshwater Research, 36(2), 177–190. 10.1071/MF9850177

[ece38730-bib-0020] Domingos, J. A. , Budd, A. M. , Banh, Q. Q. , Goldsbury, J. A. , Zenger, K. R. , & Jerry, D. R. (2018). Sex‐specific *dmrt1* and *cyp19a1* methylation and alternative splicing in gonads of the protandrous hermaphrodite barramundi. PLoS One, 13(9), e0204182. 10.1371/journal.pone.0204182 30226860 PMC6143260

[ece38730-bib-0021] Doyle, J. J. , & Doyle, J. L. (1987). Isolation of plant DNA from fresh tissue. Phytochemical Bulletin, 19(11), 11–15.

[ece38730-bib-0022] Duncan, E. J. , Cunningham, C. B. , & Dearden, P. K. (2022). Phenotypic plasticity: What has DNA methylation got to do with it? Insects, 13(2), 110. 10.3390/insects13020110 35206684 PMC8878681

[ece38730-bib-0023] Duncan, E. J. , Gluckman, P. D. , & Dearden, P. K. (2014). Epigenetics, plasticity, and evolution: How do we link epigenetic change to phenotype? Journal of Experimental Zoology Part B: Molecular and Developmental Evolution, 322(4), 208–220. 10.1002/jez.b.22571 24719220

[ece38730-bib-0024] Faircloth, B. C. , & Glenn, T. C. (2014). Faircloth‐lab serapure protocol.

[ece38730-bib-0025] Ferrari, S. , & Cribari‐Neto, F. (2004). Beta regression for modelling rates and proportions. Journal of Applied Statistics, 31(7), 799–815. 10.1080/0266476042000214501

[ece38730-bib-0026] Fisheries Queensland (2014). Fisheries long term monitoring program sampling protocol – data protocol: Appendix 3A standardised catch and sampling locations: Gulf of Carpentaria and East Coast North. Department of Agriculture, and Fisheries.

[ece38730-bib-0027] Fisheries Queensland (2020a). Biological sampling protocol: barramundi. Department of Agriculture and Fisheries.

[ece38730-bib-0028] Fisheries Queensland (2020b). Fishery monitoring – Barramundi ageing protocol. Department of Agriculture and Fisheries.

[ece38730-bib-0029] Fox, J. , Friendly, M. , & Weisberg, S. (2013). Hypothesis tests for multivariate linear models using the car package. The R Journal, 5(1), 39–52. 10.32614/RJ-2013-004

[ece38730-bib-0030] Goikoetxea, A. , Todd, E. V. , & Gemmell, N. J. (2017). Stress and sex: Does cortisol mediate sex change in fish? Reproduction, 154(6), R149–R160. 10.1530/REP-17-0408 28890443

[ece38730-bib-0031] Gorelick, N. , Hancher, M. , Dixon, M. , Ilyushchenko, S. , Thau, D. , & Moore, R. (2017). Google Earth Engine: Planetary‐scale geospatial analysis for everyone. Remote Sensing of Environment, 202, 18–27. 10.1016/j.rse.2017.06.031

[ece38730-bib-0032] Grey, D. L. (1987). An overview of *Lates calcarifer* in Australia and Asia. Management of Wild and Cultured Sea bass/barramundi, 15–21.

[ece38730-bib-0033] Guiguen, Y. , Cauty, C. , Fostier, A. , Fuchs, J. , & Jalabert, B. (1994). Reproductive cycle and sex inversion of the seabass, *Lates calcarifer*, reared in sea cages in French Polynesia: Histological and morphometric description. Environmental Biology of Fishes, 39(3), 231–247. 10.1007/BF00005126

[ece38730-bib-0034] Guo, C.‐Y. , Tseng, P.‐W. , Hwang, J.‐S. , Wu, G.‐C. , & Chang, C.‐F. (2021). Potential role of DNA methylation of cyp19a1a promoter during sex change in protogynous orange‐spotted grouper, *Epinephelus* *coioides* . General and Comparative Endocrinology, 311, 113840. 10.1016/j.ygcen.2021.113840 34216589

[ece38730-bib-0035] Han, J. , Hu, Y. , Qi, Y. , Yuan, C. , Naeem, S. , & Huang, D. (2021). High temperature induced masculinization of zebrafish by down‐regulation of sox9b and esr1 via DNA methylation. Journal of Environmental Sciences, 107, 160–170. 10.1016/j.jes.2021.01.032 34412779

[ece38730-bib-0036] Hattori, R. S. , Fernandino, J. I. , Kishii, A. I. , Kimura, H. , Kinno, T. , Oura, M. , Somoza, G. M. , Yokota, M. , Strüssmann, C. A. , & Watanabe, S. (2009). Cortisol‐induced masculinization: Does thermal stress affect gonadal fate in pejerrey, a teleost fish with temperature‐dependent sex determination? PLoS One, 4(8), e6548. 10.1371/journal.pone.0006548 19662094 PMC2717333

[ece38730-bib-0037] Hayashi, Y. , Kobira, H. , Yamaguchi, T. , Shiraishi, E. , Yazawa, T. , Hirai, T. , Kamei, Y. , & Kitano, T. (2010). High temperature causes masculinization of genetically female medaka by elevation of cortisol. Molecular Reproduction and Development, 77(8), 679–686. 10.1002/mrd.21203 20653000

[ece38730-bib-0038] Heydenrych, M. J. , Saunders, B. J. , Bunce, M. , & Jarman, S. N. (2021). Epigenetic measurement of key vertebrate population biology parameters. Frontiers in Ecology and Evolution, 9, 617376. 10.3389/fevo.2021.617376

[ece38730-bib-0039] Hu, J. , & Barrett, R. D. H. (2017). Epigenetics in natural animal populations. Journal of Evolutionary Biology, 30(9), 1612–1632. 10.1111/jeb.13130 28597938

[ece38730-bib-0040] Jerry, D. R. (2014). Biology and culture of Asian seabass Lates calcarifer. CRC Press.

[ece38730-bib-0041] Jerry, D. R. , Smith‐Keune, C. , Hodgson, L. , Pirozzi, I. , Carton, A. G. , Hutson, K. S. , Brazenor, A. K. , Trujillo Gonzalez, A. , Gamble, S. , Collins, G. , & VanDerWal, J. (2013). Vulnerability of an iconic Australian finfish (barramundi‐Lates calcarifer) and aligned industries to climate change across tropical Australia. Report. Fisheries Research and Development Corporation, Douglas, QLD, Australia.

[ece38730-bib-0042] Jombart, T. (2008). adegenet: A R package for the multivariate analysis of genetic markers. Bioinformatics, 24(11), 1403–1405. 10.1093/bioinformatics/btn129 18397895

[ece38730-bib-0043] Jones, P. A. (2012). Functions of DNA methylation: Islands, start sites, gene bodies and beyond. Nature Reviews Genetics, 13(7), 484. 10.1038/nrg3230 22641018

[ece38730-bib-0044] Katersky, R. S. , & Carter, C. G. (2005). Growth efficiency of juvenile barramundi, *Lates calcarifer*, at high temperatures. Aquaculture, 250(3), 775–780. 10.1016/j.aquaculture.2005.05.008

[ece38730-bib-0045] Kearse, M. , Moir, R. , Wilson, A. , Stones‐Havas, S. , Cheung, M. , Sturrock, S. , Buxton, S. , Cooper, A. , Markowitz, S. , Duran, C. , Thierer, T. , Ashton, B. , Meintjes, P. , & Drummond, A. (2012). Geneious Basic: An integrated and extendable desktop software platform for the organization and analysis of sequence data. Bioinformatics, 28(12), 1647–1649. 10.1093/bioinformatics/bts199 22543367 PMC3371832

[ece38730-bib-0046] Keenan, C. P. (1994). Recent evolution of population structure in Australian barramundi, *Lates calcarifer* (Bloch): An example of isolation by distance in one dimension. Marine and Freshwater Research, 45(7), 1123–1148. 10.1071/MF9941123

[ece38730-bib-0047] Kelley, J. L. , Tobler, M. , Beck, D. , Sadler‐Riggleman, I. , Quackenbush, C. R. , Arias Rodriguez, L. , & Skinner, M. K. (2021). Epigenetic inheritance of DNA methylation changes in fish living in hydrogen sulfide–rich springs. Proceedings of the National Academy of Sciences, 118(26), e2014929118. 10.1073/pnas.2014929118 PMC8255783 34185679

[ece38730-bib-0048] Kilvitis, H. J. , Hanson, H. , Schrey, A. W. , & Martin, L. B. (2017). Epigenetic potential as a mechanism of phenotypic plasticity in vertebrate range expansions. Integrative and Comparative Biology, 57(2), 385–395. 10.1093/icb/icx082 28859411

[ece38730-bib-0049] Li, L.‐C. , & Dahiya, R. (2002). MethPrimer: Designing primers for methylation PCRs. Bioinformatics, 18(11), 1427–1431. 10.1093/bioinformatics/18.11.1427 12424112

[ece38730-bib-0050] Li, S. , He, F. , Wen, H. , Li, J. , Si, Y. , Liu, M. , He, H. , & Huang, Z. (2017). Analysis of DNA methylation level by methylation‐sensitive amplification polymorphism in half smooth tongue sole (*Cynoglossus semilaevis*) subjected to salinity stress. Journal of Ocean University of China, 16(2), 269–278. 10.1007/s11802-017-3156-4

[ece38730-bib-0051] Lim, L. , Heng, H. , & Lee, H. (1986). The induced breeding of seabass, *Lates calcarifer* (Bloch) in Singapore. Singapore Journal of Primary Industries (Singapore), 14(2), 81–95.

[ece38730-bib-0052] Liu, M. , & Sadovy de Mitcheson, Y. (2011). The influence of social factors on juvenile sexual differentiation in a diandric, protogynous grouper *Epinephelus coioides* . Ichthyological Research, 58(1), 84–89. 10.1007/s10228-010-0187-x

[ece38730-bib-0053] Loughnan, S. R. , Smith‐Keune, C. , Beheregaray, L. B. , Robinson, N. A. , & Jerry, D. R. (2019). Population genetic structure of barramundi (*Lates calcarifer*) across the natural distribution range in Australia informs fishery management and aquaculture practices. Marine and Freshwater Research, 70(11), 1533. 10.1071/MF18330

[ece38730-bib-0054] Mackie, M. (2000). Reproductive biology of the halfmoon grouper, *Epinephelus rivulatus*, at Ningaloo Reef. Western Australia. Environmental Biology of Fishes, 57(4), 363–376. 10.1023/A:1007658027359

[ece38730-bib-0055] Mackie, M. C. (2003). Socially controlled sex‐change in the half‐moon grouper, *Epinephelus rivulatus*, at Ningaloo Reef, Western Australia. Coral Reefs, 22(2), 133–142. 10.1007/s00338-003-0296-3

[ece38730-bib-0056] Masser, D. R. , Berg, A. S. , & Freeman, W. M. (2013). Focused, high accuracy 5‐methylcytosine quantitation with base resolution by benchtop next‐generation sequencing. Epigenetics & Chromatin, 6, 33. 10.1186/1756-8935-6-33 24279302 PMC3907040

[ece38730-bib-0057] Massicotte, R. , & Angers, B. (2012). General‐purpose genotype or how epigenetics extend the flexibility of a genotype. Genetics Research International, 2012, 1–7. 10.1155/2012/317175 PMC3335555 22567383

[ece38730-bib-0058] Mayne, B. , Berry, O. , Davies, C. , Farley, J. , & Jarman, S. (2019). A genomic predictor of lifespan in vertebrates. Scientific Reports, 9(1), 1–10. 10.1038/s41598-019-54447-w 31831772 PMC6908713

[ece38730-bib-0059] Mayne, B. , Espinoza, T. , Roberts, D. , Butler, G. L. , Brooks, S. , Korbie, D. , & Jarman, S. (2021). Nonlethal age estimation of three threatened fish species using DNA methylation: Australian lungfish, Murray cod and Mary River cod. Molecular Ecology Resources, 21(7), 2324–2332. 10.1111/1755-0998.13440 34161658 PMC8518777

[ece38730-bib-0060] McGaughey, D. M. , Abaan, H. O. , Miller, R. M. , Kropp, P. A. , & Brody, L. C. (2014). Genomics of CpG methylation in developing and developed zebrafish. G3: Genes, Genomes. Genetics, 4(5), 861–869. 10.1534/g3.113.009514 24657902 PMC4025485

[ece38730-bib-0061] Metzger, D. C. , & Schulte, P. M. (2018). The DNA methylation landscape of stickleback reveals patterns of sex chromosome evolution and effects of environmental salinity. Genome Biology and Evolution, 10(3), 775–785. 10.1093/gbe/evy034 29420714 PMC5841383

[ece38730-bib-0062] Mohamed, A. R. , Naval‐Sanchez, M. , Menzies, M. , Evans, B. , King, H. , Reverter, A. , & Kijas, J. W. (2020). Integrated transcriptome, DNA methylome and chromatin state accessibility landscapes reveal regulators of Atlantic salmon maturation. bioRxiv. 10.1101/2020.08.28.272286

[ece38730-bib-0063] Moore, R. (1979). Natural sex inversion in the giant perch (*Lates calcarifer*). Marine and Freshwater Research, 30(6), 803–813. 10.1071/MF9790803

[ece38730-bib-0064] Morán, P. , & Pérez‐Figueroa, A. (2011). Methylation changes associated with early maturation stages in the Atlantic salmon. BMC Genetics, 12(1), 86. 10.1186/1471-2156-12-86 21982559 PMC3197556

[ece38730-bib-0065] Navarro‐Martín, L. , Viñas, J. , Ribas, L. , Díaz, N. , Gutiérrez, A. , Di Croce, L. , & Piferrer, F. (2011). DNA methylation of the gonadal aromatase (*cyp19a*) promoter is involved in temperature‐dependent sex ratio shifts in the European sea bass. PLoS Genetics, 7(12), e1002447. 10.1371/journal.pgen.1002447 22242011 PMC3248465

[ece38730-bib-0066] Newton, J. R. , Zenger, K. R. , & Jerry, D. R. (2013). Next‐generation transcriptome profiling reveals insights into genetic factors contributing to growth differences and temperature adaptation in Australian populations of barramundi (*Lates calcarifer*). Marine Genomics, 11, 45–52. 10.1016/j.margen.2013.07.002 23948424

[ece38730-bib-0067] Partridge, G. , & Lymbery, A. (2008). The effect of salinity on the requirement for potassium by barramundi (*Lates calcarifer*) in saline groundwater. Aquaculture, 278(1–4), 164–170. 10.1016/j.aquaculture.2008.03.042

[ece38730-bib-0068] Piferrer, F. , Anastasiadi, D. , Valdivieso, A. , Sánchez, N. , Moraleda, J. , & Ribas, L. (2019). The model of the conserved epigenetic regulation of sex. Frontiers in Genetics, 10, 857. 10.3389/fgene.2019.00857 31616469 PMC6775248

[ece38730-bib-0069] Pigliucci, M. (2005). Evolution of phenotypic plasticity: Where are we going now? Trends in Ecology & Evolution, 20(9), 481–486. 10.1016/j.tree.2005.06.001 16701424

[ece38730-bib-0070] Pusey, B. , Kennard, M. J. , & Arthington, A. H. (2004). Freshwater fishes of north‐eastern. CSIRO publishing.

[ece38730-bib-0071] QFMA (1991). A review of the east coast barramundi fishery and proposed management measures. Retrieved from Brisbane, Australia.

[ece38730-bib-0072] R Core Team , (2013). R: A language and environment for statistical computing.

[ece38730-bib-0073] Richards, C. L. , Bossdorf, O. , & Pigliucci, M. (2010). What role does heritable epigenetic variation play in phenotypic evolution? BioScience, 60(3), 232–237. 10.1525/bio.2010.60.3.9

[ece38730-bib-0074] Roberts, B. H. , Morrongiello, J. R. , Morgan, D. L. , King, A. J. , Saunders, T. M. , & Crook, D. A. (2021). Faster juvenile growth promotes earlier sex change in a protandrous hermaphrodite (barramundi: *Lates calcarifer*). Scientific Reports, 11(1), 1–10. 10.1038/s41598-021-81727-1 33500452 PMC7838401

[ece38730-bib-0075] Robins, J. , Mayer, D. , Staunton‐Smith, J. , Halliday, I. , Sawynok, B. , & Sellin, M. (2006). Variable growth rates of the tropical estuarine fish barramundi *Lates calcarifer* (Bloch) under different freshwater flow conditions. Journal of Fish Biology, 69(2), 379–391. 10.1111/j.1095-8649.2006.01100.x

[ece38730-bib-0076] Russell, D. J. , Thomson, F. E. , Thuesen, P. A. , Power, T. N. , & Mayer, R. J. (2015). Variability in the growth, feeding and condition of barramundi (*Lates calcarifer* Bloch) in a northern Australian coastal river and impoundment. Marine and Freshwater Research, 66(10), 928–941. 10.1071/MF13269

[ece38730-bib-0077] Seow, W. J. , Pesatori, A. C. , Dimont, E. , Farmer, P. B. , Albetti, B. , Ettinger, A. S. , Bollati, V. , Bolognesi, C. , Roggieri, P. , Panev, T. I. , Georgieva, T. , Merlo, D. F. , Bertazzi, P. A. , & Baccarelli, A. A. (2012). Urinary benzene biomarkers and DNA methylation in Bulgarian petrochemical workers: study findings and comparison of linear and beta regression models. PLoS One, 7(12), e50471. 10.1371/journal.pone.0050471 23227177 PMC3515615

[ece38730-bib-0078] Shaklee, J. B. , & Salini, J. P. (1985). Genetic variation and population subdivision in Australian barramundi, *Lates calcarifer* (Bloch). Marine and Freshwater Research, 36(2), 203–218. 10.1071/MF9850203

[ece38730-bib-0079] Shao, C. , Li, Q. , Chen, S. , Zhang, P. , Lian, J. , Hu, Q. , Sun, B. , Jin, L. , Liu, S. , Wang, Z. , Zhao, H. , Jin, Z. , Liang, Z. , Li, Y. , Zheng, Q. , Zhang, Y. , Wang, J. , & Zhang, G. (2014). Epigenetic modification and inheritance in sexual reversal of fish. Genome Research, 24(4), 604–615. 10.1101/gr.162172.113 24487721 PMC3975060

[ece38730-bib-0080] Streipert, S. , Filar, J. , Robins, J. B. , O’Neil, M. F. O. , & Whybird, O. (2019). Stock assessment of the barramundi (*Lates calcarifer*) fishery in Queensland, Australia. Department of Agriculture and Fisheries.

[ece38730-bib-0081] Stuart, I. G. , & McKillup, S. C. (2002). The use of sectioned otoliths to age barramundi (*Lates calcarifer*) (Bloch, 1790) [Centropomidae]. Hydrobiologia, 479(1–3), 231–236.

[ece38730-bib-0082] Stueve, T. R. , Wolff, M. S. , Pajak, A. , Teitelbaum, S. L. , & Chen, J. (2014). CYP19A1 promoter methylation in saliva associated with milestones of pubertal timing in urban girls. BMC Pediatrics, 14, 78. 10.1186/1471-2431-14-78 24649863 PMC4000125

[ece38730-bib-0083] Todd, E. V. , Liu, H. , Muncaster, S. , & Gemmell, N. J. (2016). Bending genders: The biology of natural sex change in fish. Sexual Development, 10(5–6), 223–241. 10.1159/000449297 27820936

[ece38730-bib-0084] Todd, E. V. , Ortega‐Recalde, O. , Liu, H. , Lamm, M. S. , Rutherford, K. M. , Cross, H. , Black, M. A. , Kardailsky, O. , Marshall Graves, J. A. , Hore, T. A. , Godwin, J. R. , & Gemmell, N. J. (2019). Stress, novel sex genes, and epigenetic reprogramming orchestrate socially controlled sex change. Science Advances, 5(7), eaaw7006. 10.1126/sciadv.aaw7006 31309157 PMC6620101

[ece38730-bib-0085] Wang, J. , Liu, Y. , Jiang, S. , Li, W. , Gui, L. , Zhou, T. , Zhai, W. , Lin, Z. , Lu, J. , & Chen, L. (2019). Transcriptomic and epigenomic alterations of Nile tilapia gonads sexually reversed by high temperature. Aquaculture, 508, 167–177. 10.1016/j.aquaculture.2019.04.073

[ece38730-bib-0086] Warner, R. R. (1988). Sex change and the size‐advantage model. Trends in Ecology & Evolution, 3(6), 133–136. 10.1016/0169-5347(88)90176-0 21227182

[ece38730-bib-0087] Wen, A. , You, F. , Sun, P. , Li, J. , Xu, D. , Wu, Z. , & Zhang, P. (2014). CpG methylation of *dmrt1* and *cyp19a* promoters in relation to their sexual dimorphic expression in the Japanese flounder *Paralichthys olivaceus* . Journal of Fish Biology, 84(1), 193–205. 24372528 10.1111/jfb.12277

[ece38730-bib-0088] Woo, N. , & Chiu, S. (1995). Effects of nitrite exposure on growth and survival of sea bass, Lates calcarifer, fingerlings in various salinities. Journal of Applied Aquaculture, 4(4), 45–54.

[ece38730-bib-0089] Wu, G.‐C. , Li, H.‐W. , Huang, C.‐H. , Lin, H.‐J. , Lin, C.‐J. , & Chang, C.‐F. (2016). The testis is a primary factor that contributes to epigenetic modifications in the ovaries of the protandrous black porgy, *Acanthopagrus schlegelii* . Biology of Reproduction, 94(6), 132, 131–113. 27103447 10.1095/biolreprod.115.137463

[ece38730-bib-0090] Yuan, C. , Zhang, C. , Qi, Y. , Li, D. , Hu, Y. , & Huang, D. (2020). 2,4‐Dichlorophenol induced feminization of zebrafish by down‐regulating male‐related genes through DNA methylation. Ecotoxicology and Environmental Safety, 189, 110042. 10.1016/j.ecoenv.2019.110042 31816500

[ece38730-bib-0091] Yue, G. H. , Xia, J. H. , Liu, F. , & Lin, G. (2012). Evidence for female‐biased dispersal in the protandrous hermaphroditic Asian seabass, *Lates* *calcarifer* . PLoS One, 7(6), e37976. 10.1371/journal.pone.0037976 22701591 PMC3373547

[ece38730-bib-0092] Zhang, Y. , Fischer, M. , Colot, V. , & Bossdorf, O. (2013). Epigenetic variation creates potential for evolution of plant phenotypic plasticity. New Phytologist, 197(1), 314–322. 10.1111/nph.12010 23121242

[ece38730-bib-0093] Zhang, Y. , Zhang, S. , Liu, Z. , Zhang, L. , & Zhang, W. (2013). Epigenetic modifications during sex change repress gonadotropin stimulation of *Cyp19a1a* in a teleost ricefield eel (*Monopterus albus*). Endocrinology, 154(8), 2881–2890. 10.1210/en.2012-2220 23744638

