# Supplementary material for: Epigenetics underpins phenotypic plasticity of protandrous sex change in fish
Source: Ecol Evol. 2022 Mar 18;12(3):e8730. doi: 10.1002/ece3.8730 (PMC8931711; doi:10.1002/ece3.8730)
Supplement: Supplementary file 1 — Supplementary Material [file ECE3-12-e8730-s001.docx]

**Supplemental Information for:**

**Epigenetics underpins phenotypic plasticity of protandrous sex change in fish**

Alyssa M. Budd^*,1,2^, Julie B. Robins^3^, Olivia Whybird^4^ and Dean R. Jerry^1,5^

^1^Centre for Sustainable Tropical Fisheries and Aquaculture, James Cook University, Townsville, Qld, 4811, Australia

^2^Centre for Tropical Bioinformatics and Molecular Biology, James Cook University, Townsville, Qld, 4811, Australia

^3^Ecosciences Precinct, Department of Agriculture and Fisheries, Brisbane, Qld 4102, Australia

^4^Northern Fisheries Centre, Department of Agriculture and Fisheries, Cairns, Qld, 4870, Australia

^5^Tropical Futures Institute, James Cook University, Singapore

Table S1 Preliminary analysis comparing the relationship between length and sex in barramundi (*Lates calcarifer*) from different regions in Queensland, Australia. The data show that there are significant differences between the Gulf of Carpentaria (GoC; n =5515) and North Qld east coast (n=1247), however, there are significant and substantial differences between the Mid-northern GoC (n=2319) and the Southern GoC (n=3169) but not the Southern GoC and the North Qld east coast. As such, the three regions (North Qld east coast, Southern GoC and Mid-northern GoC) were used for further investigation. Latitudinal coordinates and a map are provided in the main text. Simultaneous tests for general linear hypotheses were performed using multiple comparisons of means Tukey contrasts. Model fit: glm(formula = sex ~ length + region, family = binomial).

| Two regions (initial model) | | | | |
| --- | --- | --- | --- | --- |
| Linear Hypotheses: | Estimate | Std. Error | z value | Pr(>\|z\|) |
| Gulf of Carpentaria - North Qld east coast == 0 | 1.17146 | 0.08765 | 13.37 | <2e-16 *** |
| Three regions (final model) | | | | |
| Southern GoC - North Qld east coast == 0 | 0.25951 | 0.09796 | 2.649 | 0.0216 * |
| Mid-northern GoC - North Qld east coast == 0 | 2.39372 | 0.10106 | 23.687 | <0.001 *** |
| Mid-northern GoC - Southern GoC == 0 | 2.13420 | 0.07377 | 28.930 | <0.001 *** |

Figure S1. Length frequency distribution of Australian barramundi, *Lates calcarifer*, from the north Qld east coast (green), mid-northern Gulf of Carpentaria (GoC; orange) and southern GoC (blue) > 50 cm showing the total number of male (light colour shades) and female (dark colour shades) barramundi of each 10 cm size class. A. Shows all barramundi collected between 2000 and 2017 sampled for length, age and sex in each region. B. Shows only those barramundi subject to DNA methylation analysis.

Table S2 Barramundi (*Lates calcarifer*) sex-related genes and primer sequences investigated for bisulphite amplicon next-generation sequencing. Primer start and end positions are numbered in relation to the first translated base of the gene start codon (i.e., where the A in ATG is +1). Product size includes Illumina overhangs (+68 bp in total).

| Gene | Primer name | Primer sequence (5’ – 3’)^1^ | Start pos. | End pos. | Product size (bp) | Annealing (˚C) | No. CpGs |
| --- | --- | --- | --- | --- | --- | --- | --- |
| cyp19a1a | CYP19-BS-F | FO-TGGTTGTTTATAAAGGGGAAGTTT | -106 | -83 | 334 | 57.5 | 8 |
|  | CYP19-BS-R | RO-CCAACAACAAACAAACAAATAACATA | +135 | +160 |  |  |  |
| dmrt1 | D1-BS-P-F1(2) | FO-GCTGGGTGTCTTTTTACTCTCCCTGC | -30 | -4 | 271 | 57.5 | 9^2^ |
|  | D1_BS_CDS_R1 | RO-AAACACTAACAATCCCTCCAATTAC | +149 | +173 |  |  |  |
| esr1 | ESR1_BS_PE1_F1 | FO-TGTGTTGTGATGTTGTTTAGGTAGAG | -149 | -124 | 275 | 57.5 | 8 |
|  | ESR1_BS_PE1_R1 | RO-TTCCAAAAAATCCACAATAACTACC | +33 | +57 |  |  |  |
| nr5a2 | sf1-BS-F2 | FO-GGAAAAGAGATTGTTTAGTATAGTAATAGA | -75 | -46 | 261 | 57.5 | 8 |
|  | sf1-BS-R2 | RO-TAAAAACACTAACCTTACAACTCTC | +94 | +118 |  |  |  |

^1^ FO (5’ TCGTCGGCAGCGTCAGATGTGTATAAGAGACAG) and RO (5’ GTCTCGTGGGCTCGGAGATGTGTATAAGAGACAG) are Illumina’s forward overhang (FO) and reverse overhang (RO) adapter sequences added to gene-specific primer sequences.

^2^ Sequence coverage was inadequate (< 500 reads) for the first CpG site in the *dmrt1* amplicon, thus DNA methylation data are only reported for the final 8 CpG sitesFigure S2. Complete structure of barramundi (*Lates calcarifer*) sex genes indicating primer positioning used in bisulphite amplicon sequencing analyses. Diagram shows exons (blue boxes), introns (black line), primer positions (green boxes) and CpG sites (black vertical lines).

Figure S3. Structure of barramundi (*Lates calcarifer*) sex genes from -200 nt (nucleotides) to the end of the first exon indicating primer positioning used in bisulphite amplicon sequencing analyses. Diagram shows exons (blue boxes), introns (black line), primer positions (green boxes) and CpG sites (black vertical lines).

Figure S4 Correlations between HYCOM temperature values downloaded from Google Earth Engine and AIMS in situ temperature loggers for three Queensland east coast locations throughout 2019; Farquharson Reef, High Island, Lizard Island. Data were used to validate the HYCOM data for use in the regions of interest for the present study.


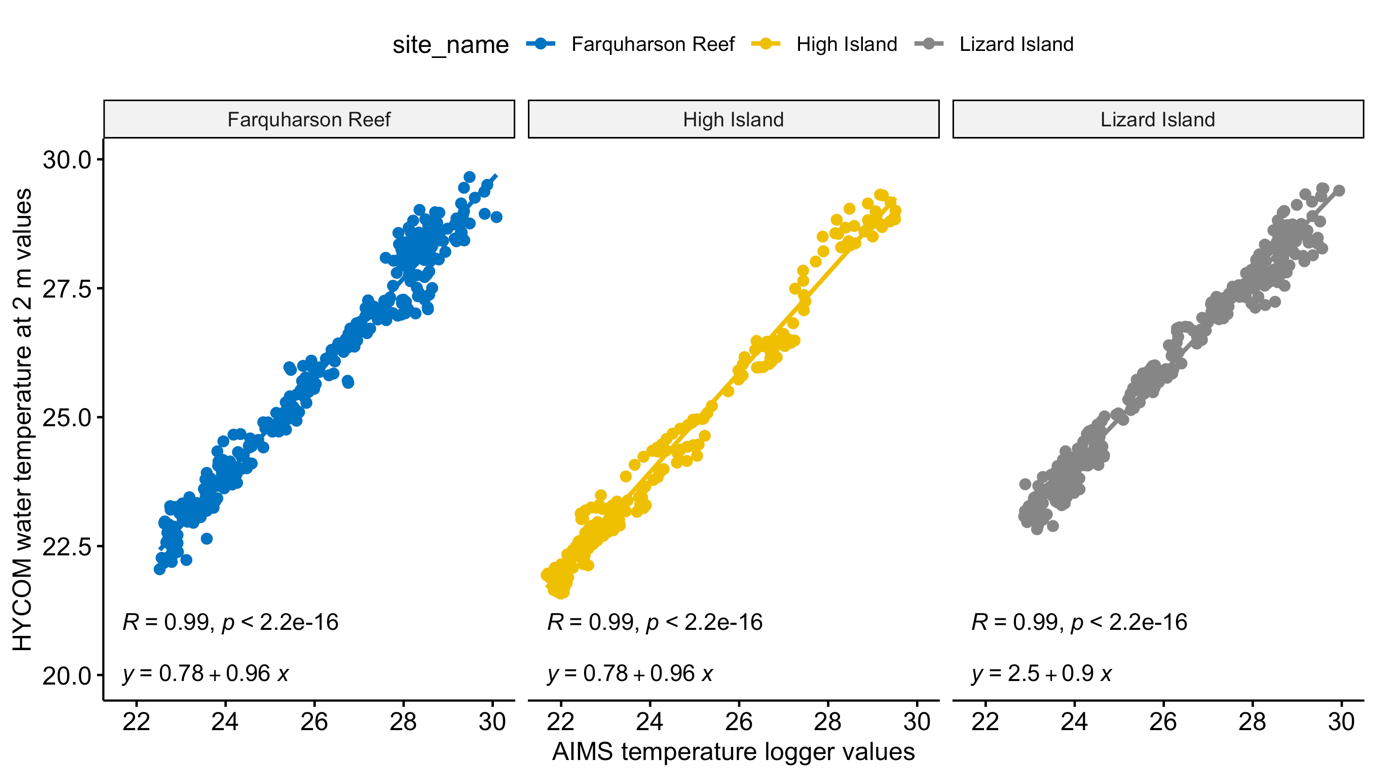


Figure S5 Fitted GLM (solid lines) with standard error (grey shading) showing predicted length at a given age for each region and weak but significant correlations between the two variables.

Table S3 Generalized variance-inflation factors (GVIF) calculated for beta regression model of proportion methylation in gonadal DNA from wild-caught *Lates calcarifer* from Queensland, Australia is modelled for amplicons of four sex-related genes using CpG site, geographic region, age and implementing the logit link function. Large GVIF values indicate greater levels of multicollinearity where values less than three are acceptable. GVIF^(1/(2*Df)) is calculated to allow comparability by reducing the GVIF to a linear measure.

| Amplicon | Covariate | Generalised VIF (GVIF) | Degrees of freedom (Df) | GVIF^(1/(2*Df)) |
| --- | --- | --- | --- | --- |
| *nr5a2* | CpG site | 1.00595358 | 1 | 1.00297237 |
|  | Region | 1.75316219 | 2 | 1.15068254 |
|  | Sex | 1.35747873 | 1 | 1.16510889 |
|  | Total length | 1.47220899 | 1 | 1.2133462 |
|  | Age | 1.69586818 | 1 | 1.30225504 |
| *esr1* | CpG site | 1.00122668 | 1 | 1.00061315 |
|  | Region | 1.73401266 | 2 | 1.1475274 |
|  | Sex | 1.31478924 | 1 | 1.14664259 |
|  | Total length | 1.44123471 | 1 | 1.20051435 |
|  | Age | 1.68235364 | 1 | 1.29705576 |
| *dmrt1* | CpG site | 1.000003 | 1 | 1.0000015 |
|  | Region | 1.82879974 | 2 | 1.16289777 |
|  | Sex | 1.35920914 | 1 | 1.16585125 |
|  | Total length | 1.46918985 | 1 | 1.21210142 |
|  | Age | 1.7125676 | 1 | 1.30865106 |
| *cyp19a1a* | CpG site | 1.03618795 | 1 | 1.01793317 |
|  | Region | 1.7651271 | 2 | 1.15264082 |
|  | Sex | 1.33384353 | 1 | 1.15492144 |
|  | Total length | 1.44832027 | 1 | 1.20346179 |
|  | Age | 1.6992645 | 1 | 1.3035584 |

Table S4 Model selection table based on Bayesian information criterion (BIC) where proportion methylation in gonadal DNA from wild-caught *Lates calcarifer* from Queensland, Australia is modelled for amplicons of four sex-related genes using beta regression with CpG site, geographic region and the covariate(s) listed in the table with all possible interaction terms and implementing the logit link function. K is the number of estimated parameters for each model. Lower BIC values indicate a better fit.

| Amplicon | Model covariate(s) | K | Log-likelihood | BIC | Delta BIC | Relative likelihood | BIC weight |
| --- | --- | --- | --- | --- | --- | --- | --- |
| *nr5a2* | Age | 23 | 548.104232 | -944.63154 | 18.275613 | 0.00010752 | 9.23E-05 |
|  | Total length | 23 | 557.242039 | -962.90715 | 0 | 1 | 0.85833473 |
|  | Total length and age | 38 | 604.867118 | -959.3028 | 3.60435755 | 0.16493913 | 0.14157298 |
| *esr1* | Age | 23 | 568.262792 | -984.94866 | 176.589863 | 4.51E-39 | 4.51E-39 |
|  | Total length | 23 | 617.955366 | -1084.3338 | 77.2047156 | 1.72E-17 | 1.72E-17 |
|  | Total length and age | 38 | 705.984982 | -1161.5385 | 0 | 1 | 1 |
| *dmrt1* | Age | 23 | 1414.28117 | -2676.9854 | 0 | 1 | 0.99930036 |
|  | Total length | 23 | 1407.01692 | -2662.4569 | 14.5284919 | 0.00070013 | 0.00069964 |
|  | Total length and age | 38 | 1434.79409 | -2619.1567 | 57.8286651 | 2.77E-13 | 2.77E-13 |
| *cyp19a1a* | Age | 23 | 871.669112 | -1591.7613 | 27.5239397 | 1.05E-06 | 1.05E-06 |
|  | Total length | 23 | 877.166631 | -1602.7563 | 16.5289024 | 0.00025751 | 0.00025744 |
|  | Total length and age | 38 | 934.85834 | -1619.2852 | 0 | 1 | 0.9997415 |

Table S5 Comparison of model covariates used to investigate DNA methylation in amplicons covering partial sex gene and promoter regions in barramundi, *Lates calcarifer*, between three geographic regions in Queensland, Australia. Results are from a Chi-squared goodness of fit (based on deviance) test showing significance of each factor variable on beta regression models with both sexes, as well as when subset by gene and gene and sex.

| Gene/Amplicon | Sex | Factor | Df | Chi-squared | FDR adjusted *p* value | |
| --- | --- | --- | --- | --- | --- | --- |
| *cyp19a1a* | Both | CpG site | 7 | 1382.784 | 0.000 | *** |
|  |  | Region | 2 | 67.599 | 0.000 | *** |
|  |  | Sex | 1 | 1765.567 | 0.000 | *** |
|  |  | Total length | 1 | 18.754 | 0.000 | *** |
|  |  | CpG site:Sex | 7 | 48.442 | 0.000 | *** |
|  |  | Region:Sex | 2 | 24.817 | 0.000 | *** |
|  |  | Region:Total length | 2 | 3.832 | 0.147 |  |
|  |  | Sex:Total length | 1 | 120.142 | 0.000 | *** |
|  |  | CpG site:Sex:Total length | 14 | 35.452 | 0.001 | ** |
|  |  | Region:Sex:Total length | 2 | 22.802 | 0.000 | *** |
|  | Female | CpG site | 7 | 477.027 | 0.000 | *** |
|  |  | Region | 2 | 44.098 | 0.000 | *** |
|  |  | Total length | 1 | 21.395 | 0.000 | *** |
|  |  | Region:Total length | 2 | 9.062 | 0.011 | * |
|  | Male | CpG site | 7 | 958.279 | 0.000 | *** |
|  |  | Region | 2 | 51.193 | 0.000 | *** |
|  |  | Total length | 1 | 191.789 | 0.000 | *** |
|  |  | Region:Total length | 2 | 16.194 | 0.000 | *** |
| *esr1* | Both | CpG site | 7 | 32.006 | 0.000 | *** |
|  |  | Region | 2 | 37.079 | 0.000 | *** |
|  |  | Sex | 1 | 474.481 | 0.000 | *** |
|  |  | Total length | 1 | 41.435 | 0.000 | *** |
|  |  | CpG site:Sex | 7 | 0.120 | 1.000 |  |
|  |  | Region:Sex | 2 | 29.547 | 0.000 | *** |
|  |  | Region:Total length | 2 | 5.625 | 0.077 | . |
|  |  | Sex:Total length | 1 | 207.238 | 0.000 | *** |
|  |  | CpG site:Sex:Total length | 14 | 0.290 | 1.000 |  |
|  |  | Region:Sex:Total length | 2 | 8.210 | 0.023 | * |
|  | Female | CpG site | 7 | 10.302 | 0.194 |  |
|  |  | Region | 2 | 36.761 | 0.000 | *** |
|  |  | Total length | 1 | 37.317 | 0.000 | *** |
|  |  | Region:Total length | 2 | 3.758 | 0.183 |  |
|  | Male | CpG site | 7 | 24.407 | 0.002 | *** |
|  |  | Region | 2 | 44.241 | 0.000 | *** |
|  |  | Total length | 1 | 364.975 | 0.000 | *** |
|  |  | Region:Total length | 2 | 11.583 | 0.005 | ** |
| *nr5a2* | Both | CpG site | 7 | 527.011 | 0.000 | *** |
|  |  | Region | 2 | 39.733 | 0.000 | *** |
|  |  | Sex | 1 | 1279.933 | 0.000 | *** |
|  |  | Total length | 1 | 218.559 | 0.000 | *** |
|  |  | CpG site:Sex | 7 | 17.786 | 0.016 | * |
|  |  | Region:Sex | 2 | 60.546 | 0.000 | *** |
|  |  | Region:Total length | 2 | 44.252 | 0.000 | *** |
|  |  | Sex:Total length | 1 | 81.116 | 0.000 | *** |
|  |  | CpG site:Sex:Total length | 14 | 5.580 | 0.976 |  |
|  |  | Region:Sex:Total length | 2 | 3.879 | 0.152 |  |
|  | Female | CpG site | 7 | 1034.809 | 0.000 | *** |
|  |  | Region | 2 | 24.707 | 0.000 | *** |
|  |  | Total length | 1 | 3.853 | 0.056 | * |
|  |  | Region:Total length | 2 | 21.173 | 0.000 | *** |
|  | Male | CpG site | 7 | 134.091 | 0.000 | *** |
|  |  | Region | 2 | 56.186 | 0.000 | *** |
|  |  | Total length | 1 | 262.177 | 0.000 | *** |
|  |  | Region:Total length | 2 | 28.047 | 0.000 | *** |
| *dmrt1* | Both | CpG site | 7 | 1178.499 | 0.000 | *** |
|  |  | Region | 2 | 41.243 | 0.000 | *** |
|  |  | Sex | 1 | 1306.356 | 0.000 | *** |
|  |  | Total length | 1 | 127.496 | 0.000 | *** |
|  |  | CpG site:Sex | 7 | 13.016 | 0.081 | . |
|  |  | Region:Sex | 2 | 105.312 | 0.000 | *** |
|  |  | Region:Total length | 2 | 27.070 | 0.000 | *** |
|  |  | Sex:Total length | 1 | 49.981 | 0.000 | *** |
|  |  | CpG site:Sex:Total length | 14 | 5.511 | 0.977 |  |
|  |  | Region:Sex:Total length | 2 | 0.714 | 0.741 |  |
|  | Female | CpG site | 7 | 987.945 | 0.000 | *** |
|  |  | Region | 2 | 98.161 | 0.000 | *** |
|  |  | Total length | 1 | 5.935 | 0.018 | * |
|  |  | Region:Total length | 2 | 17.530 | 0.000 | *** |
|  | Male | CpG site | 7 | 349.991 | 0.000 | *** |
|  |  | Region | 2 | 61.046 | 0.000 | *** |
|  |  | Total length | 1 | 203.301 | 0.000 | *** |
|  |  | Region:Total length | 2 | 11.730 | 0.004 | ** |

Table S6 Beta-regression models for methylation levels in Queensland barramundi, *Lates calcarifer*. Final models were subset be gene and sex, using the formula: Methylation ~ CpG site + region + total length + region :total length and logit link.

| Gene | **Sex** | **Beta** | **Level** | **Coefficient** | | **Z value** | **FDR Adjusted *p* value** |
| --- | --- | --- | --- | --- | --- | --- | --- |
| *cyp19a1a* | Female | (Intercept) | -83 | 3.442 | 3.569 | 0.001 | *** |
|  |  | CpG site | -52 | 0.860 | 8.839 | 0.000 | *** |
|  |  |  | -48 | 0.573 | 5.903 | 0.000 | *** |
|  |  |  | -31 | 0.446 | 4.589 | 0.000 | *** |
|  |  |  | 28 | 1.340 | 13.454 | 0.000 | *** |
|  |  |  | 38 | 0.992 | 10.161 | 0.000 | *** |
|  |  |  | 92 | 1.701 | 16.491 | 0.000 | *** |
|  |  |  | 106 | 1.613 | 15.792 | 0.000 | *** |
|  |  | Region | Mid-northern Gulf of Carpentaria | -3.409 | -3.403 | 0.001 | ** |
|  |  |  | Southern Gulf of Carpentaria | -3.414 | -3.407 | 0.001 | ** |
|  |  | Total length | Variable | -0.041 | -3.980 | 0.000 | *** |
|  |  | Region:Total length | Mid-northern Gulf of Carpentaria: variable | 0.031 | 2.860 | 0.006 | ** |
|  |  |  | Southern Gulf of Carpentaria: variable | 0.032 | 2.983 | 0.004 | ** |
|  |  | Total length + Region:Total length | Variable + Mid-northern Gulf of Carpentaria: variable | -0.010 |  |  |  |
|  |  | Total length + Region:Total length | Variable + Southern Gulf of Carpentaria: variable | -0.009 |  |  |  |
| *Pseudo R2: 0.66* | | | | | | | |
| *cyp19a1a* | Male | (Intercept) | -83 | -1.797 | -7.094 | 0.000 | *** |
|  |  | CpG site | -52 | 0.946 | 11.669 | 0.000 | *** |
|  |  |  | -48 | 0.384 | 5.197 | 0.000 | *** |
|  |  |  | -31 | 0.221 | 3.063 | 0.003 | ** |
|  |  |  | 28 | 1.911 | 18.891 | 0.000 | *** |
|  |  |  | 38 | 1.324 | 15.079 | 0.000 | *** |
|  |  |  | 92 | 2.049 | 19.566 | 0.000 | *** |
|  |  |  | 106 | 1.899 | 18.826 | 0.000 | *** |
|  |  | Region | Mid-northern Gulf of Carpentaria | 1.697 | 3.964 | 0.000 | *** |
|  |  |  | Southern Gulf of Carpentaria | -0.052 | -0.133 | 0.908 |  |
|  |  | Total length | Variable | 0.037 | 11.320 | 0.000 | *** |
|  |  | Region:Total length | Mid-northern Gulf of Carpentaria: variable | -0.022 | -3.952 | 0.000 | *** |
|  |  |  | Southern Gulf of Carpentaria: variable | -0.004 | -0.745 | 0.585 |  |
|  |  | Total length + Region:Total length | Variable + Mid-northern Gulf of Carpentaria: variable | 0.015 |  |  |  |
|  |  | Total length + Region:Total length | Variable + Southern Gulf of Carpentaria: variable | 0.033 |  |  |  |
| *Pseudo R2: 0.76* | | | | | | | |
| *Esr1* | Female | (Intercept) | -71 | 4.841 | 3.319 | 0.005 | ** |
|  |  | CpG site | -41 | 0.259 | 1.776 | 0.156 |  |
|  |  |  | -36 | 0.254 | 1.739 | 0.164 |  |
|  |  |  | -29 | 0.299 | 2.048 | 0.096 | . |
|  |  |  | -20 | 0.360 | 2.454 | 0.040 | * |
|  |  |  | -18 | 0.337 | 2.300 | 0.057 | . |
|  |  |  | 8 | 0.400 | 2.722 | 0.019 | * |
|  |  |  | 18 | 0.359 | 2.447 | 0.040 | * |
|  |  | Region | Mid-northern Gulf of Carpentaria | -3.006 | -1.986 | 0.103 |  |
|  |  |  | Southern Gulf of Carpentaria | -3.388 | -2.239 | 0.061 | . |
|  |  | Total length | Variable | -0.048 | -3.074 | 0.009 | ** |
|  |  | Region:Total length | Mid-northern Gulf of Carpentaria: variable | 0.024 | 1.465 | 0.277 |  |
|  |  |  | Southern Gulf of Carpentaria: variable | 0.030 | 1.870 | 0.131 |  |
|  |  | Total length + Region:Total length | Variable + Mid-northern Gulf of Carpentaria: variable | -0.024 |  |  |  |
|  |  | Total length + Region:Total length | Variable + Southern Gulf of Carpentaria: variable | -0.017 |  |  |  |
| *Pseudo R2: 0.18* | | | | | | | |
| *Esr1* | Male | (Intercept) | -71 | -3.181 | -10.470 | 0.000 | *** |
|  |  | CpG site | -41 | 0.311 | 2.961 | 0.011 | * |
|  |  |  | -36 | 0.317 | 3.017 | 0.009 | ** |
|  |  |  | -29 | 0.347 | 3.296 | 0.005 | ** |
|  |  |  | -20 | 0.405 | 3.820 | 0.001 | ** |
|  |  |  | -18 | 0.374 | 3.542 | 0.002 | ** |
|  |  |  | 8 | 0.435 | 4.084 | 0.000 | *** |
|  |  |  | 18 | 0.381 | 3.605 | 0.002 | ** |
|  |  | Region | Mid-northern Gulf of Carpentaria | 1.854 | 3.591 | 0.002 | ** |
|  |  |  | Southern Gulf of Carpentaria | -0.231 | -0.484 | 0.999 |  |
|  |  | Total length | Variable | 0.058 | 14.626 | 0.000 | *** |
|  |  | Region:Total length | Mid-northern Gulf of Carpentaria: variable | -0.023 | -3.240 | 0.005 | ** |
|  |  |  | Southern Gulf of Carpentaria: variable | -0.001 | -0.157 | 0.999 |  |
|  |  | Total length + Region:Total length | Variable + Mid-northern Gulf of Carpentaria: variable | 0.035 |  |  |  |
|  |  | Total length + Region:Total length | Variable + Southern Gulf of Carpentaria: variable | 0.057 |  |  |  |
| *Pseudo R2: 0.52* | | | | | | | |
| *nr5a2* | Female | (Intercept) | -30 | 1.401 | 2.360 | 0.055 | . |
|  |  | CpG site | -19 | 0.519 | 8.445 | 0.000 | *** |
|  |  |  | 20 | 0.027 | 0.440 | 0.987 |  |
|  |  |  | 32 | 0.010 | 0.161 | 0.987 |  |
|  |  |  | 44 | -0.440 | -6.957 | 0.000 | *** |
|  |  |  | 50 | 0.760 | 12.244 | 0.000 | *** |
|  |  |  | 77 | 0.984 | 15.592 | 0.000 | *** |
|  |  |  | 92 | 1.112 | 17.418 | 0.000 | *** |
|  |  | Region | Mid-northern Gulf of Carpentaria | -2.109 | -3.412 | 0.003 | ** |
|  |  |  | Southern Gulf of Carpentaria | -1.060 | -1.714 | 0.204 |  |
|  |  | Total length | Variable | -0.017 | -2.603 | 0.029 | * |
|  |  | Region:Total length | Mid-northern Gulf of Carpentaria: variable | 0.022 | 3.190 | 0.005 | ** |
|  |  |  | Southern Gulf of Carpentaria: variable | 0.009 | 1.347 | 0.379 |  |
|  |  | Total length + Region:Total length | Variable + Mid-northern Gulf of Carpentaria: variable | 0.005 |  |  |  |
|  |  | Total length + Region:Total length | Variable + Southern Gulf of Carpentaria: variable | -0.008 |  |  |  |
| *Pseudo R2: 0.82* | | | | | | | |
| *nr5a2* | Male | (Intercept) | -30 | 2.940 | 9.176 | 0.000 | *** |
|  |  | CpG site | -19 | 0.318 | 2.749 | 0.020 | * |
|  |  |  | 20 | 0.034 | 0.287 | 0.987 |  |
|  |  |  | 32 | 0.028 | 0.236 | 0.987 |  |
|  |  |  | 44 | -0.272 | -2.222 | 0.072 | . |
|  |  |  | 50 | 0.515 | 4.522 | 0.000 | *** |
|  |  |  | 77 | 0.650 | 5.749 | 0.000 | *** |
|  |  |  | 92 | 0.719 | 6.386 | 0.000 | *** |
|  |  | Region | Mid-northern Gulf of Carpentaria | -3.092 | -5.707 | 0.000 | *** |
|  |  |  | Southern Gulf of Carpentaria | -0.556 | -1.121 | 0.494 |  |
|  |  | Total length | Variable | -0.058 | -14.069 | 0.000 | *** |
|  |  | Region:Total length | Mid-northern Gulf of Carpentaria: variable | 0.038 | 5.296 | 0.000 | *** |
|  |  |  | Southern Gulf of Carpentaria: variable | 0.012 | 1.857 | 0.155 |  |
|  |  | Total length + Region:Total length | Variable + Mid-northern Gulf of Carpentaria: variable | -0.020 |  |  |  |
|  |  | Total length + Region:Total length | Variable + Southern Gulf of Carpentaria: variable | -0.046 |  |  |  |
| *Pseudo R2: 0.48* | | | | | | | |
| *dmrt1* | Female | (Intercept) | -5 | -0.210 | -0.395 | 0.880 |  |
|  |  | CpG site | 8 | -0.383 | -4.569 | 0.000 | *** |
|  |  |  | 32 | 1.087 | 16.488 | 0.000 | *** |
|  |  |  | 39 | 0.745 | 10.896 | 0.000 | *** |
|  |  |  | 77 | 0.918 | 13.700 | 0.000 | *** |
|  |  |  | 97 | 1.003 | 15.098 | 0.000 | *** |
|  |  |  | 111 | -0.051 | -0.653 | 0.771 |  |
|  |  |  | 142 | -0.044 | -0.571 | 0.798 |  |
|  |  | Region | Mid-northern Gulf of Carpentaria | -2.557 | -4.570 | 0.000 | *** |
|  |  |  | Southern Gulf of Carpentaria | -1.904 | -3.398 | 0.002 | ** |
|  |  | Total length | Variable | -0.022 | -3.850 | 0.000 | *** |
|  |  | Region:Total length | Mid-northern Gulf of Carpentaria: variable | 0.024 | 3.919 | 0.000 | *** |
|  |  |  | Southern Gulf of Carpentaria: variable | 0.016 | 2.621 | 0.028 | * |
|  |  | Total length + Region:Total length | Variable + Mid-northern Gulf of Carpentaria: variable | 0.002 |  |  |  |
|  |  | Total length + Region:Total length | Variable + Southern Gulf of Carpentaria: variable | -0.006 |  |  |  |
| *Pseudo R2: 0.81* | | | | | | | |
| *dmrt1* | Male | (Intercept) | -5 | -0.510 | -2.042 | 0.109 |  |
|  |  | CpG site | 7 | -0.331 | -3.014 | 0.008 | ** |
|  |  |  | 31 | 0.886 | 9.899 | 0.000 | *** |
|  |  |  | 38 | 0.562 | 6.012 | 0.000 | *** |
|  |  |  | 76 | 0.658 | 7.130 | 0.000 | *** |
|  |  |  | 96 | 0.696 | 7.585 | 0.000 | *** |
|  |  |  | 110 | -0.129 | -1.223 | 0.443 |  |
|  |  |  | 141 | -0.183 | -1.712 | 0.198 |  |
|  |  | Region | Mid-northern Gulf of Carpentaria | -1.757 | -3.956 | 0.000 | *** |
|  |  |  | Southern Gulf of Carpentaria | -0.402 | -1.054 | 0.551 |  |
|  |  | Total length | Variable | -0.038 | -11.996 | 0.000 | *** |
|  |  | Region:Total length | Mid-northern Gulf of Carpentaria: variable | 0.020 | 3.369 | 0.003 | ** |
|  |  |  | Southern Gulf of Carpentaria: variable | 0.009 | 1.731 | 0.197 |  |
|  |  | Total length + Region:Total length | Variable + Mid-northern Gulf of Carpentaria: variable | -0.018 |  |  |  |
|  |  | Total length + Region:Total length | Variable + Southern Gulf of Carpentaria: variable | -0.030 |  |  |  |
| *Pseudo R2: 0.60* | | | | | | | |

Table S7 Comparison of DNA methylation in amplicons covering partial sex gene and promoter regions in barramundi, *Lates calcarifer*, between three geographic regions in Queensland, Australia. Results are derived from linear hypothesis testing to compare regression coefficients for each region specified in the comparison column based on the beta regression model specified in text and *p* values are corrected based on false discovery rate (FDR) methods of Benjamini & Hochberg (1995). Results show that regression coefficients and therefore methylation levels are significantly different in the mid-northern gulf compared to both other study regions in male barramundi for all four amplicons investigated and female barramundi for *nr5a2*. Df = degrees of freedom.

| Sex | Amplicon | Residual df | Df | Comparison (null hypothesis) | Comparison coefficients | Chi-square statistic | FDR adjusted *p* value |
| --- | --- | --- | --- | --- | --- | --- | --- |
| Male | *cyp19a1a* | 426 | 1 | southern GoC = mid-northern GoC | -0.0522 = 1.6965 | 14.2776 | 5e-04*** |
|  |  |  |  | southern GoC = north Qld east coast | -1.7487 = -1.6965 | 0.0176 | 0.9332 |
|  |  |  |  | mid-northern GoC = north Qld east coast | 1.7487 = 0.0522 | 15.7142 | 3e-04*** |
|  | *dmrt1* | 426 | 1 | southern GoC = mid-northern GoC | -0.4018 = -1.757 | 8.0728 | 0.0077** |
|  |  |  |  | southern GoC = north Qld east coast | 1.3553 = 1.757 | 1.1102 | 0.3504 |
|  |  |  |  | mid-northern GoC = north Qld east coast | -1.3553 = 0.4018 | 15.6529 | 3e-04*** |
|  | *esr1* | 426 | 1 | southern GoC = mid-northern GoC | -0.2309 = 1.8542 | 13.5347 | 7e-04*** |
|  |  |  |  | southern GoC = north Qld east coast | -2.0851 = -1.8542 | 0.2343 | 0.6855 |
|  |  |  |  | mid-northern GoC = north Qld east coast | 2.0851 = 0.2309 | 12.8918 | 9e-04*** |
|  | *nr5a2* | 426 | 1 | southern GoC = mid-northern GoC | -0.5556 = -3.0923 | 18.5584 | 1e-04*** |
|  |  |  |  | southern GoC = north Qld east coast | 2.5367 = 3.0923 | 1.257 | 0.3312 |
|  |  |  |  | mid-northern GoC = north Qld east coast | -2.5367 = 0.5556 | 32.5645 | 0*** |
| Female | *cyp19a1a* | 274 | 1 | southern GoC = mid-northern GoC | -3.4138 = -3.4093 | 1.00E-04 | 0.9908 |
|  |  |  |  | southern GoC = north Qld east coast | -0.0045 = 3.4093 | 11.6048 | 0.0013** |
|  |  |  |  | mid-northern GoC = north Qld east coast | 0.0045 = 3.4138 | 11.5778 | 0.0013** |
|  | *dmrt1* | 274 | 1 | southern GoC = mid-northern GoC | -1.9043 = -2.5568 | 6.2843 | 0.0195* |
|  |  |  |  | southern GoC = north Qld east coast | 0.6525 = 2.5568 | 11.548 | 0.0013** |
|  |  |  |  | mid-northern GoC = north Qld east coast | -0.6525 = 1.9043 | 20.8876 | 1e-04*** |
|  | *esr1* | 274 | 1 | southern GoC = mid-northern GoC | -3.388 = -3.0063 | 0.4252 | 0.5878 |
|  |  |  |  | southern GoC = north Qld east coast | -0.3817 = 3.0063 | 5.0145 | 0.0377* |
|  |  |  |  | mid-northern GoC = north Qld east coast | 0.3817 = 3.388 | 3.9449 | 0.0664. |
|  | *nr5a2* | 274 | 1 | southern GoC = mid-northern GoC | -1.0598 = -2.1089 | 17.516 | 2e-04*** |
|  |  |  |  | southern GoC = north Qld east coast | 1.049 = 2.1089 | 2.9392 | 0.1153 |
|  |  |  |  | mid-northern GoC = north Qld east coast | -1.049 = 1.0598 | 11.6397 | 0.0013** |

Table S8 Comparison of DNA methylation levels between male and female barramundi, *Lates calcarifer*, from Queensland, Australia by amplicon. IQR = interquartile range.

| Amplicon | *cyp19a1a* | *dmrt1* | *esr1* | *nr5a2* |
| --- | --- | --- | --- | --- |
| Mann Whitney U (W) | 10833 | 108650 | 19870 | 113500 |
| Z | -18.89506 | -1.63E+01 | -15.63031 | -18.03144 |
| r | 3.636356 | 3.13321 | 3.008054 | 3.470152 |
| Male median methylation (IQR) | 0.91 (0.16) | 0.04 (0.04) | 0.83 (0.19) | 0.22 (0.22) |
| Female median methylation (IQR) | 0.59 (0.27) | 0.11 (0.10) | 0.59 (0.26) | 0.52 (0.23) |
| Difference (male-female median methylation) | 0.33 | -0.07 | 0.24 | -0.29 |
| *p* value | 6.26e-80 | 6.77e-60 | 2.26e-55 | 5.52e-73 |

Figure S6 Proportion of methylation in male (left column) and female (right column) *Lates calcarifer* explained by age and CpG position (indicated by symbol) for amplicons of male-associated amplicons *nr5a2 and dmrt1* and female associated amplicons *cyp19a1a* and *esr1*. Fitted curves correspond to beta regression with logit link for three regions in Queensland, Australia (indicated by colour). Model: Proportion methylated ~ CpG site + region + age + region :age with logit link.

Figure S7 Comparison of DNA methylation levels between male and female barramundi (*Lates calcarifer*) from wild caught individuals captured in north Queensland, Australia as part of a previous study (Domingos et al., 2018). Box plots demonstrate sex-specific DNA methylation in *nr5a2,* but not *sox9.* Letters denote significant differences between males and female resulting from ANOVA. Both amplicons span the start codon, where the *nr5a2* amplicon spans -75 bp to +118 bp, and the sox9 amplicon spans -129 bp to +214.
